# MOF Glass Confined Black Phosphorus via Co─P Anchoring for Advanced Lithium‐Ion Battery Anodes

**DOI:** 10.1002/advs.202511772

**Published:** 2025-08-20

**Authors:** Yijie Wei, Zhengjie Chen, Xin Guo, Huixian Xie, Zhefei Sun, Sahar Osman, Jun Xiao, Tianyu Chen, Kwan San Hui, Hui‐Ming Cheng, Kwun Nam Hui

**Affiliations:** ^1^ Joint Key Laboratory of the Ministry of Education Institute of Applied Physics and Materials Engineering University of Macau Avenida da Universidade, Taipa Macau SAR 999078 P. R. China; ^2^ Faculty of Materials Science and Energy Engineering Shenzhen University of Advanced Technology Shenzhen 518055 P. R. China; ^3^ Institute of Technology for Carbon Neutrality Shenzhen Institutes of Advanced Technology Chinese Academy of Sciences Shenzhen 518055 P. R. China; ^4^ State Key Laboratory of Physical Chemistry of Solid Surfaces College of Materials Xiamen University Xiamen 361005 P. R. China; ^5^ Department of Mechanical Engineering College of Engineering Prince Mohammad Bin Fahd University P.O. Box 1664 Al Khobar 31952 Saudi Arabia

**Keywords:** anode materials, black phosphorus, heterostructure, lithium‐ion batteries, MOF glass

## Abstract

Although lithium‐ion batteries (LIBs) dominate the commercial energy storage market, the prevailing graphite anode is approaching its theoretical capacity limit. Alloy‐type anode materials like black phosphorus (BP) offer high theoretical capacity and intrinsic conductivity, but suffer from severe volume expansion and resultant structural instability. Here, an in situ vitrification strategy is reported to construct a composite anode material by integrating BP, Ketjenblack, and single wall carbon nanotube into a zeolitic imidazolate framework (ZIF) glass matrix. The established 3D network provides rapid electron and Li^+^ transport pathways, while BP nanoparticles are strongly anchored to Co nodes within the disordered ZIF glass via Co─P bonding. This architecture facilitates the pre‐activation of deeply embedded Li⁺ storage sites of ZIF glass and effectively buffers volume changes of BP, limiting structural expansion to less than 10%, as evidenced by in situ TEM. As a result, the as‐fabricated composite anode delivers a high reversible capacity of 652.3 mAh g^−1^ with ≈98% capacity retention over 1000 cycles at 1 A g^−1^. This work demonstrates the potential of metal–organic framework (MOF) glass as a robust matrix to stabilize alloy‐type anode materials, offering a promising avenue for the development of next‐generation LIB anode materials.

## Graphical abstract

A composite anode material inspired by the “Fast Train Crossing Building” architecture is designed and fabricated, which integrates a carbon conductive network and black phosphorus (BP) anchored to metal–organic framework (MOF) glass via Co─P bonding. This structure enables rapid electron/ion transport and pre‐activates deep lithium storage sites, resulting in a multi‐fold performance enhancement compared to pristine MOF crystal and glass anodes.

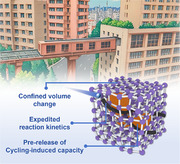

## 1 Introduction

Lithium‐ion batteries (LIBs) are pivotal to modern energy storage systems, underpinning applications ranging from portable electronics, electric vehicles (EVs), and grid‐scale storage systems, due to their high energy density, long cycle life, and proven reliability. However, the conventional graphite anode with a low theoretical capacity of 372 mA h g^−1^ resulting from its intercalation mechanism, presents a significant bottleneck—particularly for high energy applications such as long‐range EVs.^[^ ^1^ ^]^ Alloy‐type anode materials, like phosphorus (P), have attracted increasing attention owing to their high theoretical capacity enabled by multi‐electron alloying reactions.^[^ ^2^, ^3^, ^4^, ^5^ ^]^ Black phosphorus (BP), the most thermodynamically stable P allotrope with high theoretical capacity (2596 mAh g^−1^), favorable electronic conductivity, and distinctive layered structure, shows great promise for high‐energy and fast‐charging LIBs.^[^ ^6^, ^7^ ^]^ Nevertheless, BP undergoes substantial volumetric expansion of over 300% during lithiation, leading to structural degradation, electrode pulverization, and rapid capacity fading.^[^ ^8^, ^9^ ^]^ Although nanostructuring and heterostructure engineering strategies have been explored to mitigate these challenges, achieving stable long‐term cycling life remains elusive.^[^ ^10^, ^11^ ^]^

Metal–organic frameworks (MOFs), assembled from metal ions and organic ligands, exhibit highly tunable pore structures, versatile kinds, and customizable topologies delivering desired electrochemical properties such as controllable Li^+^ migration channels and abundant storage sites.^[^ ^12^, ^13^, ^14^, ^15^, ^16^ ^]^ However, MOFs confront some chief challenges, from generally low conductivity to disruption of structure causing rapid capacity decay.^[^ ^17^, ^18^, ^19^, ^20^ ^]^ As a favorable method to improve the electrochemical performance of MOFs, vitrification can be applied to generate MOF glass.^[^ ^21^, ^22^, ^23^ ^]^ MOF glass combines the tunable structure of crystalline MOFs with the advantages of glasses (e.g., isotropic properties, compositional flexibility), lacking grain boundaries and presenting a disordered open structure.^[^ ^24^, ^25^, ^26^ ^]^ Disordered MOF glass can efficiently shorten the ion diffusion distance and offer an open structure that mitigates mechanical stress, accelerating reaction kinetics and conducing cycling stability.^[^ ^27^, ^28^, ^29^, ^30^ ^]^ A subset of MOFs, cobalt‐ZIF‐62 glass, was first examined as an anode material for LIBs verifying that upon vitrification and cycling, additional channels are released for ion diffusion and storage owing to the increased distortions and local breakage of the Co─N coordination bonds, which enables the cycling‐induced increase of capacity.^[^ ^31^ ^]^ Despite certain enhancements of their performance, inferior rate performance, relatively low reversible capacity, and long cycling‐activation process until fully releasing active sites that restrict their practical application, need to be improved.^[^ ^32^ ^]^

Herein, we report a composite anode material fabricated via in situ vitrification, in which BP nanoparticles and a Ketjenblack/single‐walled carbon nanotube (SWCNT) conductive network are embedded into a ZIF‐derived glass matrix. In this composite structure, BP is encapsulated within the ZIF glass matrix and strongly anchored to its cobalt sites via Co─P bonding to form a stable BP/CoP heterostructure, while the carbon network interweaves through the glass to establish continuous electron and ion transport pathways.^[^ ^33^ ^]^ This configuration not only facilitates rapid electrochemical kinetics and pre‐activates deeply embedded storage sites, but also significantly buffers the volume changes of BP. As a result, the composite exhibits an ultralow volume variation (3.5% after delithiation) and delivers substantially enhanced capacity and long‐term cycling stability, offering a new strategy for engineering high‐performance alloy/MOF glass hybrid anode materials for next‐generation LIBs.

## 2 Results and Discussion

**Scheme 1:**
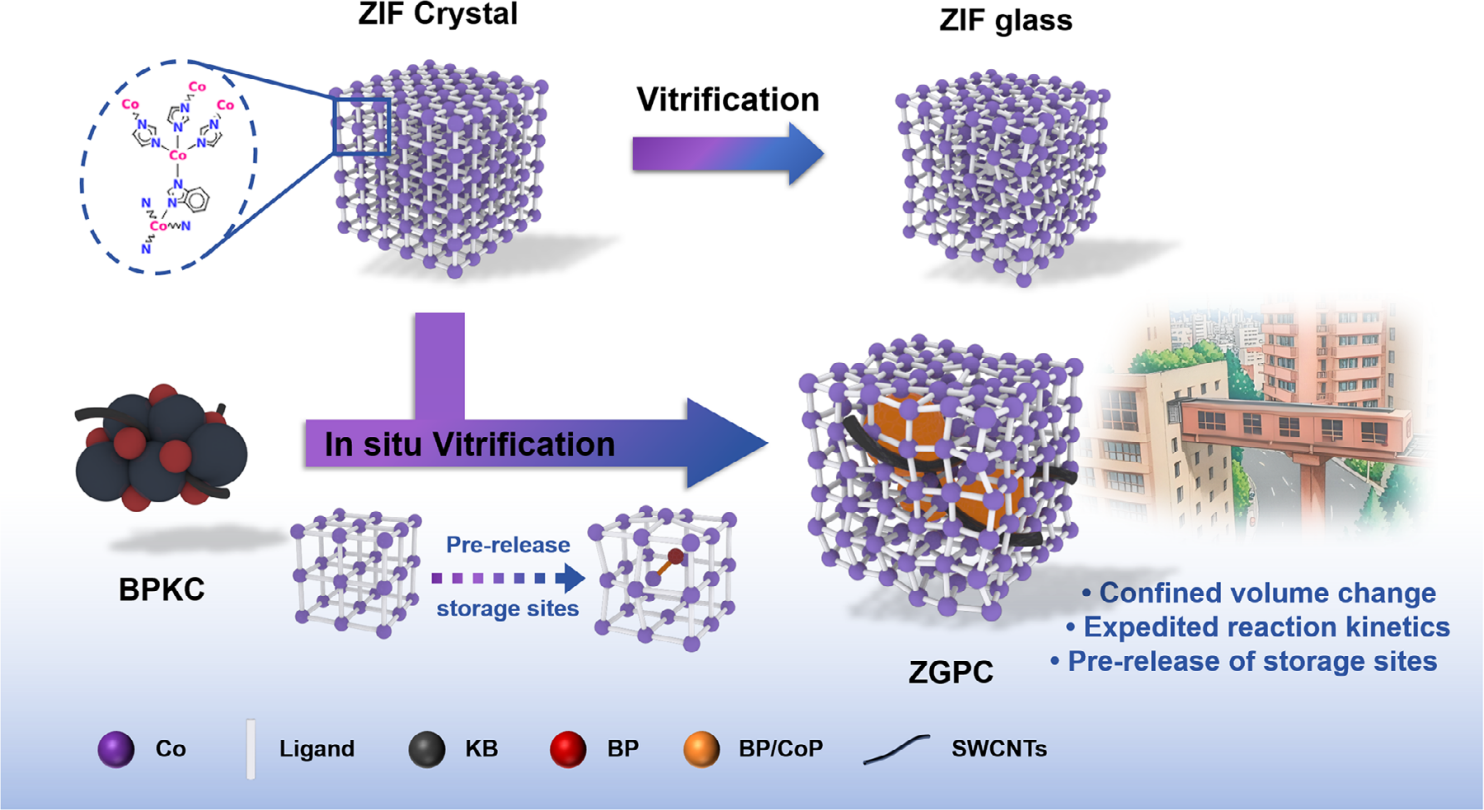
Schematic of the preparation process of ZGPC and its structural advantages as an anode material for LIBs.

**Scheme** **1** illustrates the synthesis route and structural advantages of the targeted composite material for LIBs. Cobalt‐ZIF‐62 crystals (Co(Im)_1.75_(bIm)_0.25_, denoted as ZC) were synthesized via a conventional solvothermal method, and subsequently transformed into ZIF glass (ZG) by a melt‐quenching process.^[^ ^31^, ^34^ ^]^ BP, Ketjenblack, and SWCNT were first subjected to high‐energy ball milling, generating a uniform mixture referred to as BPKC.^[^ ^35^ ^]^ The target material (ZIF glass/BPKC composite, denoted as ZGPC) was then fabricated by mixing ZC with BPKC followed by in situ vitrification via the same melt‐quenching treatment.

This synthesis strategy fabricates a ZGPC composite that translates structural design into functional electrochemical advantages. Its configuration is reminiscent of the iconic 3D “Fast Train Crossing Building” in Chongqing, China, with three key structural features: 1) The long‐range disorder coupled with preserved short‐range order in ZG resembles the interwoven building blocks of the mountain city. This disordered yet permeable framework facilitates ion transport by shortening diffusion pathways and accommodating volume changes—akin to managing “passenger crowds” in a confined, dynamic space. 2) The Ketjenblack‐SWCNT network serves as “fast train railways,” enabling rapid electron transport and pre‐establishing continuous pathways for ion and electron migration. 3) BP is robustly anchored to the ZG matrix through the formation of Co─P bonds during vitrification, which activates additional lithium‐accessible sites—functioning as a “high‐capacity station” that supports high lithium flux during charging/discharging. Altogether, this architecture‐inspired design yields a synergistic effect to improve lithium storage performance.

**Figure 1:**
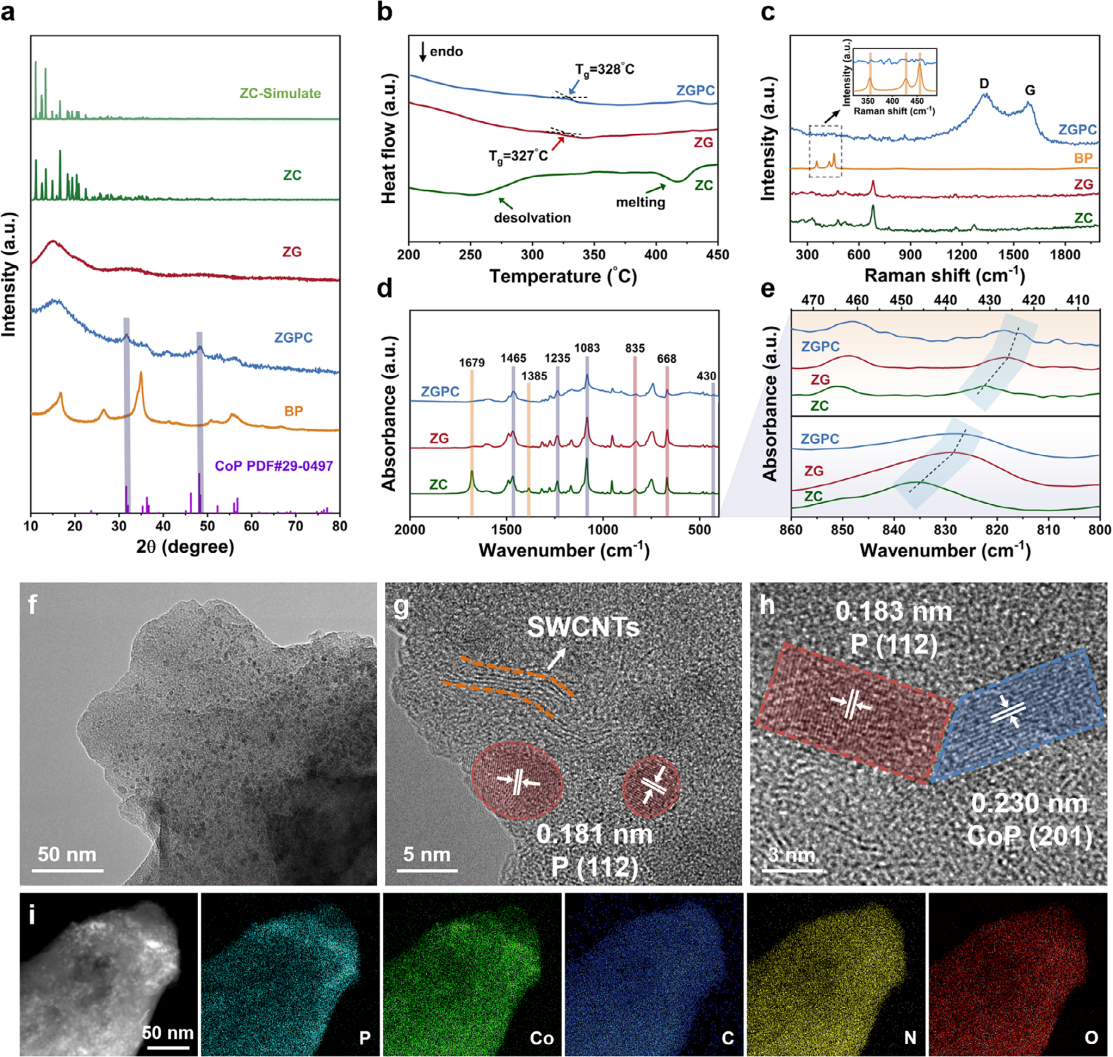
a) XRD patterns of simulated ZC and as‐synthesized ZC, ZG, ZGPC, BP, and standard CoP. b) DSC upscan curves obtained at 10 °C min^−1^ in argon. c) Raman spectra in the range from 200 to 2000 cm^−1^ (Inset: high‐resolution spectra from 320 to 490 cm^−1^). d) FTIR spectra of ZC, ZG, and ZGPC. e) Enlarged views of FTIR spectra within the frequency range of 400–480 cm^−1^ (top) and 800–860 cm^−1^ (bottom). f) TEM image, g,h) HRTEM images, and i) EDS mapping of ZGPC.

The crystallographic information of the as‐prepared samples was characterized by X‐ray diffraction (XRD). As displayed in **Figure** **1**a, the as‐synthesized ZC exhibits a similar XRD pattern to the simulated pattern of ZIF‐62 (CCDC No. 1849817), verifying the success of synthesizing ZIF‐62 crystals. After melt‐quenching, sharp diffraction peaks associated with ZC disappear in both ZG and ZGPC, demonstrating their transition to an amorphous state. Notably, ZGPC exhibits additional peaks at ≈35.0 and 55.8 degrees, corresponding to the (111) and (151) planes of BP (PDF#73‐1358). Furthermore, peaks at 31.6° and 48.4° are indexed to the CoP phase (PDF#29‐0497), indicating that during in situ vitrification with BPKC, Co sites in ZG interact with BP to form CoP/BP heterostructures. This implies strong anchoring of BP within the glass matrix via Co─P bonding in ZGPC.

Differential scanning calorimetry (DSC) and thermogravimetric analysis (TGA) were conducted to investigate the phase transitions and thermal stability of the samples (Figure 1b; Figure S1, Supporting Information). ZC exhibits two endothermic peaks at ≈260 and 420 °C, referring to solvent removal and melting of the crystalline ZIF, respectively. In contrast, both ZG and ZGPC display typical glass transition behavior during heating, with glass transition temperatures (T_g_) determined to be 327 and 328 °C, respectively. These results are in good agreement with the features of melt‐quenched glass and demonstrate that the glass nature of the framework is preserved in ZGPC after in situ vitrification with BPKC.^[^ ^34^ ^]^ Besides, the TGA curve of BP shows its thermal stability up to ≈450 °C, beyond which vaporization begins.^[^ ^36^ ^]^ Based on this, the vaporization temperature was set at 430 °C—sufficient to induce melting of ZC while below the vaporization point of BP—ensuring successful vitrification without losing active BP component.

The Raman spectrum (Figure 1c) of ZG coincides with that of ZC, indicating that the organic ligands remain chemically intact after vitrification.^[^ ^34^ ^]^ In the case of ZGPC, the featured peaks associated with the organic ligands are still present, while additional peaks emerge at 366, 439, and 466 cm^−1^, ascribing to the A1 g, B 2 g, and A2 g vibration modes of BP, respectively.^[^ ^10^, ^37^ ^]^ Moreover, two broad peaks centered at 1345 and 1590 cm^−1^ are observed, which can be attributed to the D and G bands of the Ketjenblack‐SWCNT conductive network.^[^ ^35^, ^38^ ^]^

Figure 1d presents the Fourier‐transform infrared (FTIR) spectra of ZC, ZG, and ZGPC samples. Notably, two peaks observed in ZC at 1385 and 1679 cm^−1^, corresponding to C─H vibrations and the carbonyl groups of residual DMF solvent respectively, are absent in both ZG and ZGPC.^[^ ^23^, ^38^ ^]^ This change verifies that the solvent removal occurs during the melt‐quenching treatment, in agreement with the DSC and TG results. Aside from this difference, all three samples exhibit similar characteristic peaks at ≈430, 1083, 1235, and 1465 cm^−1^, which are ascribed to Co─N bonds, C─H out‐of‐plane‐bending, C─H in‐plane‐bending, and C─N stretching, respectively.^[^ ^34^ ^]^ Two additional peaks at 668 and 835 cm^−1^ correspond to out‐of‐plane ring deformation and in‐plane bending of aromatic rings.^[^ ^31^, ^32^ ^]^ These results confirm that the organic ligands are largely preserved after the vitrification with BPKC.^[^ ^23^, ^34^, ^39^ ^]^ The enlarged FTIR spectra in Figure 1e show that the peaks at ≈430 and 835 cm^−1^ undergo subtle redshifts in ZG and ZGPC, suggesting a weakening of Co─N bonds and aromatic ring interactions upon vitrification.^[^ ^24^, ^31^, ^32^ ^]^

The morphology of samples was examined by a scanning electron microscope (SEM) and a transmission electron microscope (TEM). As shown in Figure S2 (Supporting Information), ZC and ZG display an angular block‐like morphology, while ZGPC exhibits a quasi‐spherical shape. TEM imaging of ZGPC (Figure 1f) reveals numerous dark dots dispersed in the bulk matrix, which are identified as BP nanoparticles based on high‐resolution TEM (HRTEM) analyses (Figure 1g,h; Figure S3, Supporting Information). SWCNT are also observed within the amorphous ZIF glass, positioned adjacent to the BP nanoparticles. Additionally, BP particles are largely anchored to Co sites in the glass matrix in the form of CoP, with clear phase boundaries and distinctive lattice fringes corresponding to the crystalline planes of BP and CoP, confirming the formation of BP/CoP heterostructures.^[^ ^40^ ^]^ Energy‐dispersive X‐ray spectroscopy (EDS) mapping from both TEM and SEM (Figure 1i; Figure S4, Supporting Information) further confirms the presence and uniform distribution of key elements (P, Co, C, N, and O), indicating the successful integration of composite components.

**Figure 2:**
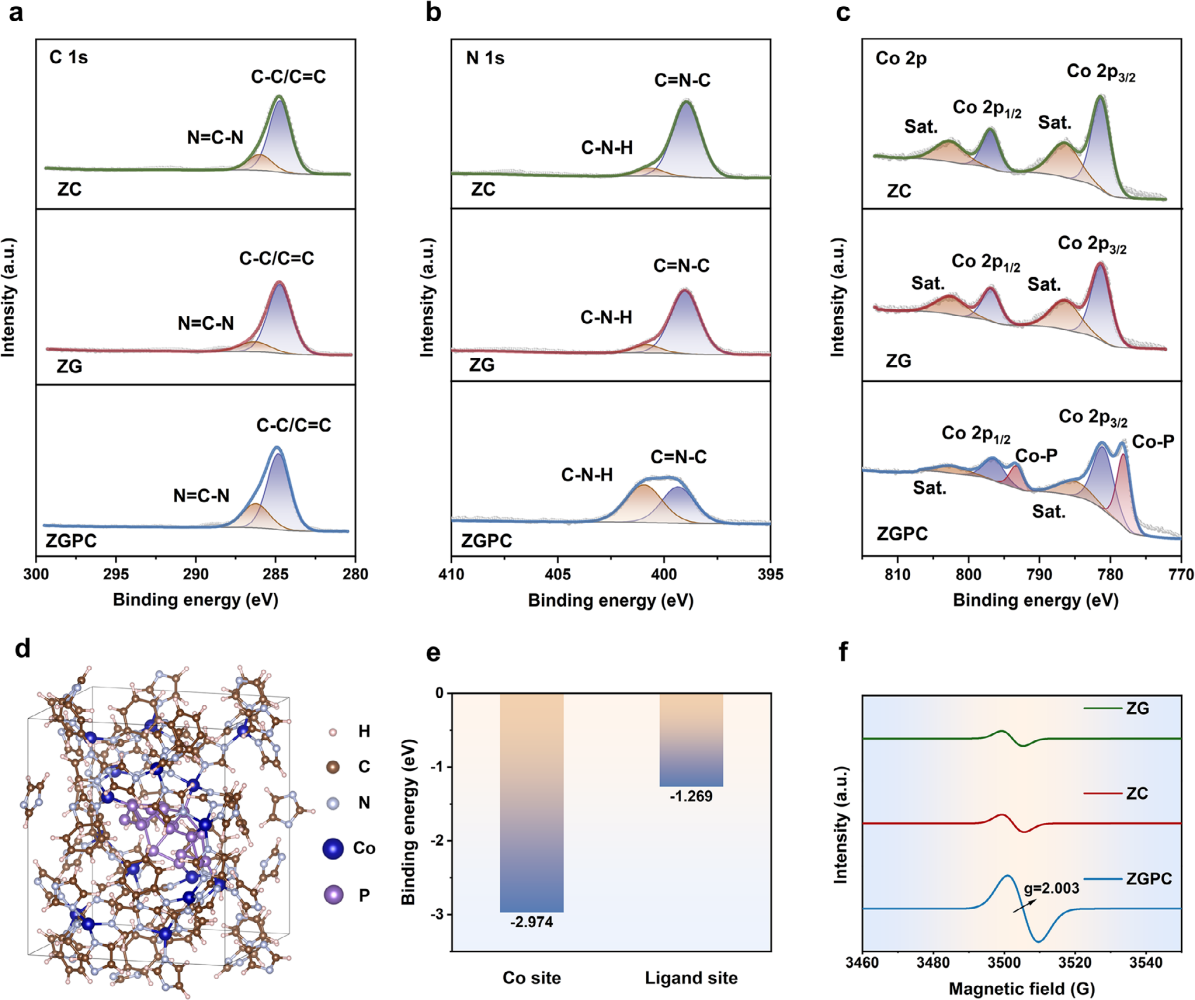
High resolution XPS spectra of a) C 1s, b) N 1s, and c) Co 2p for ZC, ZG, and ZGPC. d) The optimized configuration of BP/CoP heterostructure in ZIF (BP/CoP@ZIF) and e) the calculated binding energies of BP with a Co site and Ligand site of ZC. f) EPR spectra of ZC, ZG, and ZGPC.

X‐ray photoelectron spectroscopy (XPS) was performed to probe the surface chemistry of the prepared samples (Figure S5, Supporting Information). The ZGPC presents comparable spectral profiles to ZG and ZC in both C 1s and N 1s spectra, indicating the retention of imidazole and benzimidazole ligands after vitrification (**Figure** **2**a,b). However, a noticeable decrease in the intensity of the C═N─C peak is observed in ZGPC, indicating a reduced proportion of this bonding motif compared to ZC and ZG. The reduction mirrors the trend seen in the N 1s spectra of ZC and ZG electrodes after extended cycling in LIBs, where the C═N─C contribution decreases from 89.3% to 78.1% in ZC and from 88.5% to 75.8% in ZG (Figure S6 and Table S1, Supporting Information). These variations can be attributed to partial depolymerization of the tetrahedral Co(Im/bIm)_4_ network caused by Li^+^ (de)intercalation, which exposes more Li^+^ accessible sites.^[^ ^31^, ^32^ ^]^ Accordingly, the reduced fraction of C═N─C in ZGPC may similarly arise from partial depolymerization, likely triggered by the interaction between Co nodes and BP during vitrification, thereby pre‐activating the buried lithium storage sites within the glassy matrix.

Further evidence is provided by the Co 2p XPS spectrum of ZGPC (Figure 2c), which exhibits distinctive peaks at 793.3 and 778.1 eV attributed to Co─P bonding.^[^ ^41^, ^42^, ^43^, ^44^, ^45^ ^]^ These peaks are absent in ZC, ZG, and the control samples of ZCP and ZGP (prepared by physically mixing BP with ZC and ZG; see Figures S7 and S8, Supporting Information), verifying that Co─P bond formation is unique to the in situ vitrification process and indicative of strong BP anchoring at Co sites. In‐depth P 2p analysis provides additional insights (Figure S9, Supporting Information). On the surface, all samples show P─O signals, consistent with the high susceptibility of BP to oxidation upon air exposure.^[^ ^46^, ^47^ ^]^ However, after Ar^+^ etching, signals corresponding to P 2p_3/2_ and P 2p_1/2_ become increasingly apparent in ZGPC, whereas they nearly disappear in ZCP and ZGP. These findings suggest that, in ZCP and ZGP, BP is predominantly located on the surface of the ZIF structures, while BP in ZGPC is encapsulated within the glass matrix due to in situ vitrification. This encapsulation is expected to buffer the volumetric changes of BP during lithiation/delithiation and enhance the structural stability of BP during repeated lithiation and delithiation.

To understand the formation mechanism of BP/CoP heterostructures, density functional theory (DFT) calculations were conducted. Figure 2d showcases the optimized structural configuration of a BP/CoP heterostructure within ZIF (denoted as BP/CoP@ZIF). The binding energies calculation results in Figure 2e reveal that BP binds much more strongly to a Co site (−2.974 eV) than to a ligand site (−1.269 eV), confirming that Co nodes act as preferred anchoring sites for BP, thereby driving the formation of BP/CoP heterostructures. During the formation process, the weak coordination bonds in the ZIF framework are susceptible to distortion or cleavage, leading to partial depolymerization of the framework and generation of structural defects. Electron paramagnetic response (EPR) spectra further support this assumption (Figure 2f; Figure S10, Supporting Information), where ZGPC shows a significantly enhanced signal with a *g*‐value of 2.003, suggesting a much higher concentration of paramagnetic centers—primarily defects and oxygen vacancies—than the other samples. These defects are known to facilitate both Li⁺ adsorption and ion diffusion kinetics, thereby boosting electrochemical performance.^[^ ^48^, ^49^ ^]^ The N_2_ adsorption/desorption isotherms and pore size distribution analyses (Figure S11, Supporting Information) disclose that ZGPC achieves a higher Brunauer–Emmett–Teller (BET) surface area (34.1 m^2^ g^−1^) than ZC and ZG (<7.0 m^2^ g^−1^), along with an enlarged average pore diameter of 18.4 nm. This increased porosity could be ascribed to the integration of multi‐dimensional components into the ZGPC, as well as the establishment of additional diffusion and storage channels associated with the formation of BP/CoP heterostructures. Overall, while the primary ZIF skeleton remains intact, its partial structural reconfiguration during vitrification gives rise to enlarged ion transport pathways and defect‐mediated storage sites, which are expected to improve the lithium storage performance.

**Figure 3:**
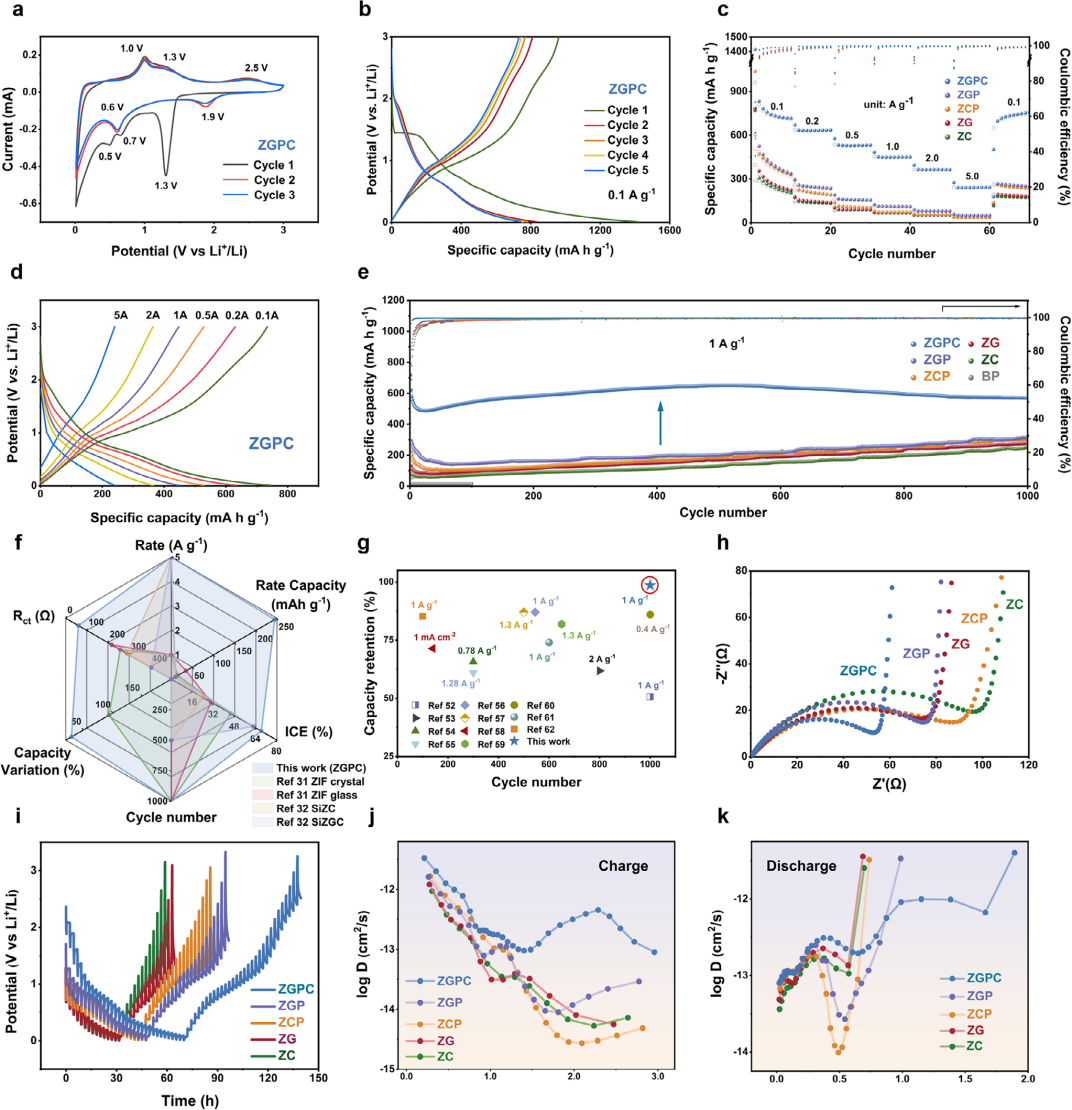
a) CV curves of ZGPC within the range of 0.01–3.0 V at a scan rate of 0.1 mV s^−1^. b) GCD profiles of ZGPC at 0.1 A g^−1^ for the initial 5 cycles. c) Rate performance of the prepared electrode material. d) Selected GCD profiles of ZGPC at various current densities. e) Long cycling performance of the prepared electrode material at 1 A g^−1^. f) Radar plots comparing key performance metrics of the ZGPC with other MOF glass‐based anode materials for LIBs. g) Cycling performance comparison of ZGPC with recently reported phosphorus‐based anode materials for LIBs. h) Nyquist plots of the fresh electrodes obtained in the frequency range of 0.01 to 100 kHz with an amplitude of 10 mV. i) GITT profiles of as‐prepared samples and the relationship between j) charge and k) discharge voltages and the corresponding Li^+^ diffusion coefficients.

To evaluate the electrochemical performance of the prepared anode materials, we assembled CR2032‐type coin cells using lithium metal foil as the counter electrode and 1 M LiPF_6_ in EC/DEC (1:1 vol%) as the electrolyte. **Figure** **3**a exhibits the cyclic voltammetry (CV) curves of ZGPC, where a distinct reduction peak at ≈1.3 V appears during the first cathodic scan, corresponding to the conversion reaction of $\mathrm{CoP}\;+\;3Li^{+}\;+\;3e^{-}\;\to \;\mathrm{Co}\;+\;Li_{3}P$.^[^ ^50^, ^51^ ^]^ Two additional cathodic peaks at ≈0.7 and 0.5 V are attributed to the stepwise lithiation of BP. In the subsequent anodic scan, a sharp peak at 1.0 V and a broad shoulder peak at 1.3 V correspond to the delithiation processes of BP and ZIF structure, respectively, while a smaller peak at ≈2.5 V is likely derived from the delithiation of CoP. In the following cycles, the CV profiles display good reproducibility, with merged lithiation peaks for BP and ZIF (≈0.6 V) and stabilized CoP redox couples (≈1.9 V for reduction and 2.5 V for oxidation), confirming good electrochemical reversibility. In contrast, the CV curves of the control samples show inferior reversibility, while no redox peaks associated with the CoP conversion reaction are observed in ZCP and ZGP (Figure S12, Supporting Information), further validating that the CoP phase forms uniquely in ZGPC through in situ vitrification.

Figure 3b presents the first five galvanostatic charge–discharge (GCD) cycles of ZGPC anode material at 0.1 A g^−1^. The composite delivers a high initial reversible capacity of 962.5 mAh g^−1^, surpassing the counterparts (Figure S13, Supporting Information). In the 1^st^ cycle, discharge plateaus at ≈1.3 V and charge plateaus at 1.0–1.25 V are consistent with redox features observed in CV curves, while the nearly overlapping profiles from 2–5 cycles verify its excellent reversibility. Figure 3c,d show the rate performance of the prepared electrodes. Compared to the control samples, ZGPC delivers superior average specific discharge capacities of 814.4, 637.6, 535.7, 453.3, 367.6, and 244.6 mAh g^−1^ at current densities of 0.1, 0.2, 0.5, 1, 2, and 5 A g^−1^, respectively. Notably, upon the current returning to 0.1 A g^−1^, the capacity recovers to over 800 mAh g^−1^, demonstrating outstanding structural stability and rate performance.

Figure 3e exhibits the long‐term cycling performance of the electrodes at 1 A g^−1^. The ZGPC presents good stability with a remarkable 98% capacity retention (compared to the reversible capacity at the third cycle) after 1000 cycles. Notably, ZGPC maintains high capacity for only the initial several cycles; and the control samples show a gradual capacity increase upon cycling, prolonged activation behavior typically observed in ZIF and ZIF glass‐based material. Although ZCP and ZGP deliver high initial discharge capacities of 576.1 and 719.2 mAh g^−1^ owing to the addition of BP, their capacities sharply decline to 182.7 and 261.3 mAh g^−1^ in the early cycles, similar to the fading trend of pure BP. In the following cycles, their capacities converge with those of the ZC and ZG, indicating that simple mechanical mixing and surface distribution of BP on ZIF are insufficient to prevent BP pulverization and stabilize the electrode structure. It is found that ZGPC strikingly outperforms the reported MOF glass‐based anode materials in the key aspects of electrochemical performance for LIBs (Figure 3f; Table S2, Supporting Information) and delivers extraordinary cyclic stability compared to recent phosphorus/phosphide‐based anode materials (Figure 3g; Table S3, Supporting Information).^[^ ^52^, ^53^, ^54^, ^55^, ^56^, ^57^, ^58^, ^59^, ^60^, ^61^, ^62^ ^]^ The excellent cycling performance of ZGPC can be assigned to its unique architecture, in which BP nanoparticles are both encapsulated and strongly anchored within the ZIF glass matrix via Co─P bonding. This structure not only enables efficient utilization of BP, pre‐constructs ion transport channels, provides additional lithium storage sites, but also buffers volume changes during cycling. Furthermore, an optimized BP content of 20% in the composite (Figure S14, Supporting Information) ensures a balanced enhancement of both capacity and cycling stability.

To investigate the charge storage mechanism, CV curves at different scan rates were collected (Figure S15, Supporting Information). By analyzing the relationship between current response and scan rate, the relative contributions of capacitive‐controlled and diffusion‐controlled processes were quantitatively determined for each electrode material. As shown in Figure S16 (Supporting Information), the ZGPC presents the highest capacitive contribution ratio among all prepared materials, which is beneficial for electrochemical kinetics and likely accounts for the superior rate capability.^[^ ^63^ ^]^

Electrochemical impedance spectroscopy (EIS) measurements were performed along with cycling tests to examine the charge transfer kinetics of the as‐prepared materials (Figure 3h; Figure S17, Supporting Information). The corresponding fitted results, obtained from the equivalent circuit models (Figure S18, Supporting Information), are summarized in Table S4 (Supporting Information). The ZGPC before and after cycling exhibits significantly lower charge transfer resistance (R_ct_) than those of the other electrodes. Moreover, both ZG and ZGP show reduced R_ct_ values compared to ZC and ZCP, indicating that the disordered structure of ZIF glass facilitates improved charge transport.

Furthermore, the lithium ion diffusion coefficients (D_Li_^+^) were calculated using the galvanostatic intermittent titration technique (GITT) (Figure 3i–k), where ZGPC exhibits consistently higher D_Li_^+^ values in the range of 10^−11^ to 10^−13^ cm^2^ s^−1^ than other samples. This indicates accelerated lithium‐ion diffusion kinetics. In addition, the activation energies (E_a_) of the materials were analyzed using Arrhenius plots derived from temperature‐dependent charge transfer resistance measurements before and after cycling (Figure S19, Supporting Information). ZGPC shows the lowest E_a_ in both cases, suggesting more favorable electrochemical reaction kinetics. Moreover, ZGPC exhibits enhanced electrolyte wettability (Figure S20, Supporting Information), evidenced by a lower contact angle of 19.0° with instant spreading behavior, superior to BP (30.3°) and BPKC (23.3°). This electrolyte‐philic character reduces the polarization and facilitates Li^+^ diffusion in ZGPC, directly contributing to the improved rate performance and further underscoring its superiority over the control samples. These results together confirm the superior electrochemical kinetics of ZGPC stemmed from the synergistic integration of the Ketjenblack‐SWCNT conductive network, the BP/CoP heterostructure, and the disordered ZIF glass framework.

**Figure 4:**
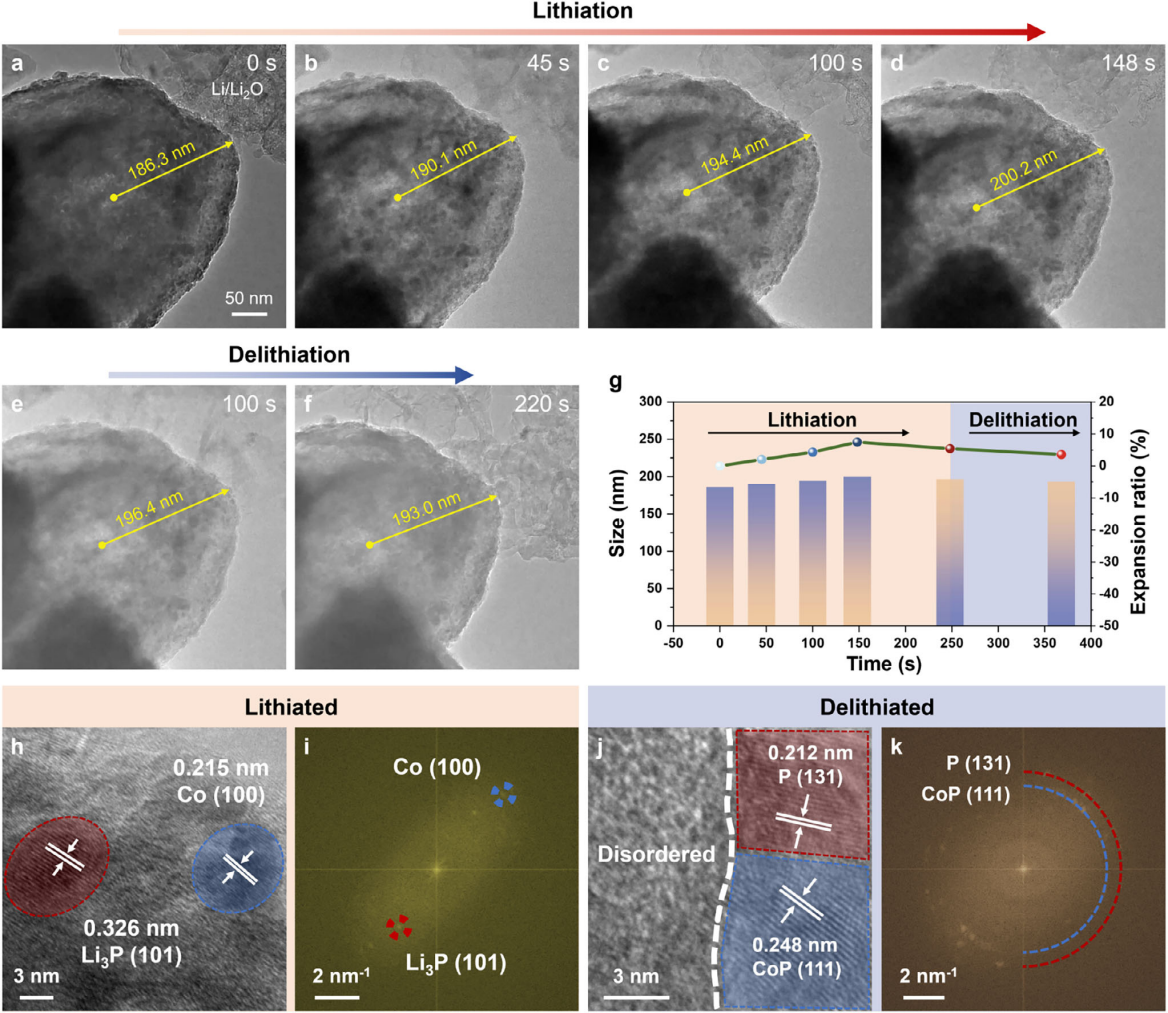
In situ TEM images showing the a–d) lithiation and e,f) delithiation process of ZGPC. g) Calculated volume variations of ZGPC during cycling. HRTEM image and corresponding FFT analyses of ZGPC at h,i) lithiated and j,k) delithiated state.

To explore the structural evolution and the Li^+^ storage mechanism of the ZGPC anode material during lithiation/delithiation, we conducted in situ TEM observations (**Figure** **4**a–f; Movie S1, Supporting Information). The ZGPC undergoes an impressively ultralow volume expansion of merely 7.5% upon full lithiation (Figure 4g), which is tremendously lower than the theoretical 300% expansion of pure BP.^[^ ^64^ ^]^ HRTEM images and corresponding selected area electron diffraction (SAED) analyses (Figure 4h,i; Figure S21, Supporting Information) reveal the lithiation products as Li_3_P and metallic Co, indicating the occurrence of alloying reactions with BP and conversion reactions with CoP. During the subsequent delithiation process, the structure reversibly shrinks with only 3.5% variation in dimension. Post‐delithiation imaging (Figure 4j,k) reveals the reappearance of distinct BP and CoP phases with well‐defined interfacial boundaries, suggesting the reversible regeneration of the BP/CoP heterostructures. Ex situ SEM was further used to track morphological evolutions of the material during long‐term cycling (Figure S22, Supporting Information). Initially, all pristine electrode materials exhibit compact surfaces with a well‐preserved particle morphology. However, after 500 and 1000 cycles, severe structural degradation is observed in ZCP and ZGP, where the active materials become fragmented due to the unrestrained volume expansion of BP during lithiation. In stark contrast, the ZGPC retains its structural integrity, characterized by nanotextured, moss‐like particles. This exceptional structural resilience supports both volume expansion mitigation and enhanced ion transport, which collectively contribute to the outstanding cycling stability and remarkable rate performance of ZGPC.

**Figure 5:**
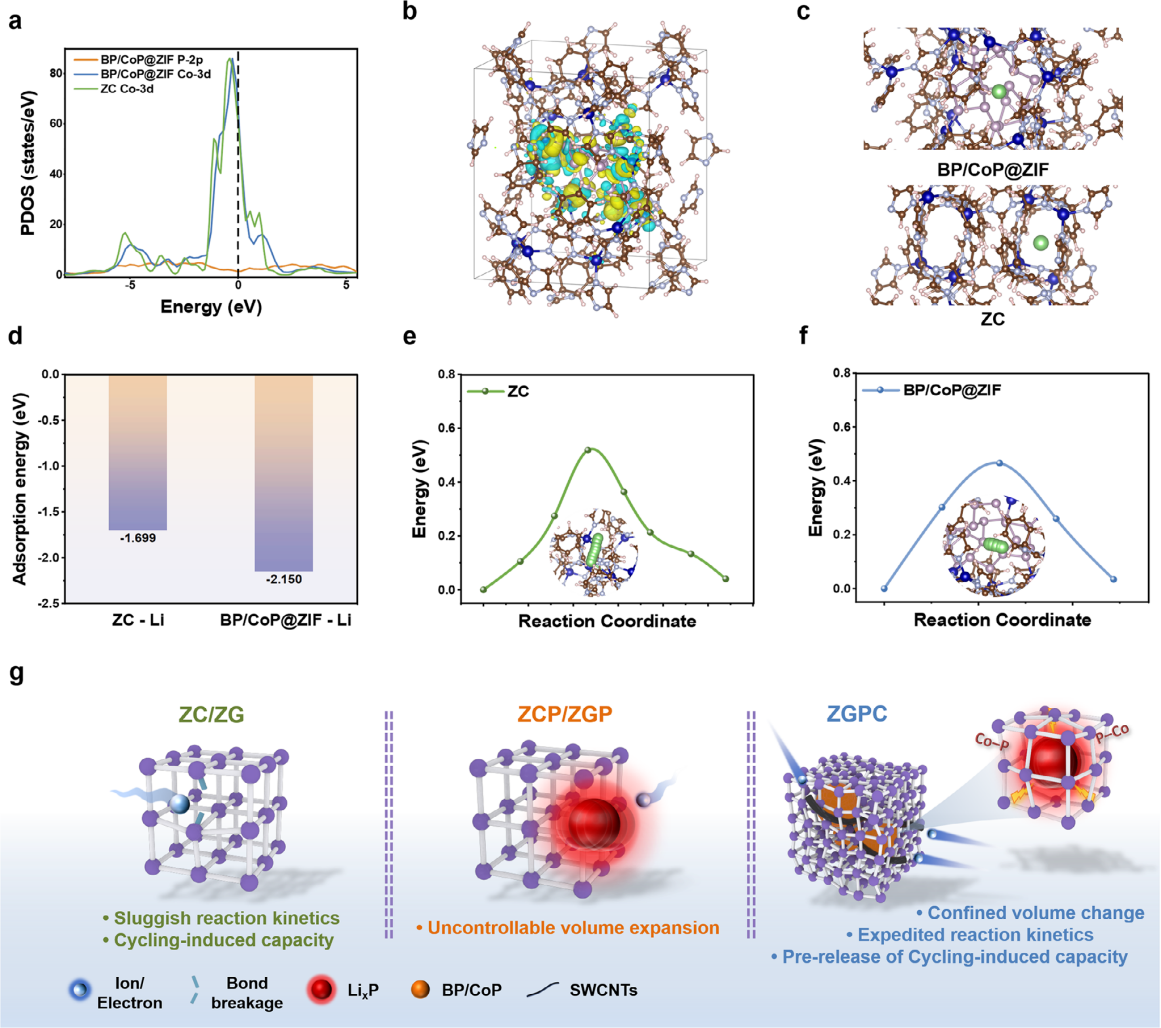
a) PDOS curves of ZC and BP/CoP@ZIF. The charge density differences within BP/CoP@ZIF b) with the yellow and blue regions referring to charge depletion and aggregation. c) Schematic of Li^+^ adsorption sites on ZC and BP/CoP@ZIF, and d) Li^+^ adsorption energy of the proposed configurations. The corresponding diffusion energy barriers for Li^+^ diffusion in e) ZC and f) BP/CoP@ZIF. g) Schematic describing the potential mechanism of the advantages of ZGPC as an anode material for LIBs.

DFT calculations were carried out based on the optimized configurations of the ZIF framework (Figure S23, Supporting Information) to gain deep insights into the electronic structure and Li^+^ storage behavior of ZGPC. The projected density of states (PDOS) for ZC and BP/CoP@ZIF is depicted in **Figure** **5**a. Compared to ZC, BP/CoP@ZIF exhibits stronger electron delocalization around the Fermi energy level, which can be ascribed to the built‐in electric field established at the interface. This internal field is expected to promote charge carrier mobility.^[^ ^40^, ^65^ ^]^ Figure 5b presents the charge difference distribution of the BP/CoP heterostructure within the ZIF matrix, where the observed charge separation further supports the presence of a strong internal electric field. Li⁺ adsorption behavior was also investigated according to the configurations shown in Figure 5c and Figure S24 (Supporting Information). BP/CoP@ZIF demonstrates a remarkably enhanced Li^+^ adsorption energy of −2.150 eV, compared to −1.699 eV for ZC, indicating a stronger interaction with Li^+^ and thus greater uptake capability during the electrochemical cycling (Figure 5d). Additionally, BP/CoP@ZIF shows a lower Li^+^ diffusion energy barrier of 0.46 than 0.52 eV for ZC (Figure 5e,f; Figure S25, Supporting Information), suggesting more efficient Li^+^ migration and accelerated reaction kinetics of the ZGPC architecture.

The advances of the designed composite material for LIBs in comparison with the pristine ZIF crystal and glass as well as mechanically mixed samples are summarized in Figure 5g. Unlike the ZC and ZG, which rely on the gradual breakage of coordination bonds during cycling to progressively activate capacity, ZGPC demonstrates a remarkable pre‐release of cycling‐induced capacity buried in ZIF glass. This enhancement originates from the early exposure of deeply embedded storage sites within the disordered ZIF glass matrix. Moreover, ZGPC enables superior utilization of BP, attributed to the strong anchoring interaction between BP and Co sites. This anchoring effect not only stabilizes BP but also allows the disordered structure to effectively buffer volume changes and mitigate the uncontrolled expansion typically associated with BP‐based anode material. Furthermore, the integration of the Ketjenblack–SWCNT conductive network and the BP/CoP heterostructures synergistically enhances both ion and electron transport, thereby accelerating reaction kinetics. Collectively, the composite material of ZGPC—reminiscent of the “Fast Train Crossing Building”—generates a strong synergistic effect, leading to exceptional lithium storage performance.

## 3 Conclusion

We have developed a ZGPC composite by integrating black phosphorus conductive carbon hybrid (BPKC) into a ZIF glass matrix through an in situ vitrification strategy. The resulting architecture features a robust BP/CoP heterostructure, enabled by strong Co─P bonding, and a continuous Ketjenblack–SWCNT conductive network throughout the matrix, which together promote fast ion/electron transport and structural stability. The disordered ZIF glass framework not only buffers volume expansion of BP but also facilitates lithium diffusion by exposing deep‐lying storage sites. As a result, the ZGPC delivers over fivefold and threefold increases in average specific discharge capacity over the first 1000 cycles at 1 A g^−1^ of the pristine ZC and ZG, respectively. The effects of structural confinement, interfacial anchoring, and conductive network engineering synergistically endow the ZGPC with superior rate capability, high Coulombic efficiency, and long‐term cycling stability. This work highlights a promising strategy to unlock the electrochemical potential of MOF‐derived glasses and offers a versatile design concept for advanced anode materials in next‐generation lithium‐ion batteries.

## Conflict of Interest

The authors declare no conflict of interest.

## Supporting information



Supporting Information

Supplemental Movie 1

## Data Availability

The data that support the findings of this study are available from the corresponding author upon reasonable request.
