# Supplementary material for: MOF Glass Confined Black Phosphorus via Co─P Anchoring for Advanced Lithium‐Ion Battery Anodes
Source: Adv Sci (Weinh). 2025 Aug 20;12(43):e11772. doi: 10.1002/advs.202511772 (PMC12631851; doi:10.1002/advs.202511772)
Supplement: Supplementary file 1 — Supporting Information [file ADVS-12-e11772-s001.docx]

Supporting Information

**MOF Glass Confined Black Phosphorus via Co–P Anchoring for Advanced Lithium-Ion Battery Anodes**

*Y**ijie Wei, Zhengjie Chen, Xin Guo,* Huixian Xie, Zhefei Sun, Sahar Osman, Jun Xiao, Tianyu Chen, Kwan San Hui, Hui-Ming Cheng, and Kwun Nam Hui**

Y. Wei, H. Xie, T. Chen, K. N. Hui

Joint Key Laboratory of the Ministry of Education, Institute of Applied Physics and Materials Engineering, University of Macau, Avenida da Universidade, Taipa, Macau SAR, 999078 China

E-mail: bizhui@um.edu.mo

Y. Wei, Z. Chen, X. Guo, H.M. Cheng

Faculty of Materials Science and Energy Engineering, Shenzhen University of Advanced Technology, Shenzhen, 518055 P. R. China

E-mail: x.guo1@siat.ac.cn

S. Osman, J. Xiao, H.M. Cheng

Institute of Technology for Carbon Neutrality, Shenzhen Institutes of Advanced Technology, Chinese Academy of Sciences, Shenzhen, 518055 P. R. China

Z. Sun

State Key Laboratory of Physical Chemistry of Solid Surfaces, College of Materials, Xiamen University, Xiamen, 361005 P. R. China

K. S. Hui

Department of Mechanical Engineering, College of Engineering, Prince Mohammad Bin Fahd University, P.O. Box 1664, Al Khobar, 31952 Kingdom of Saudi Arabia

**Experimental Section**

**Material Synthesis:** Black phosphorus (BP) was obtained from red phosphorus (RP) (Aladdin, 99.99%) via ball milling using the HEBM machine (FRITSCH Pulverisette-7 planetary ball mill from Germany) for 16 h at 1000 rpm in a stainless steel jar under an atmosphere of argon. Ketjenblack (ECP-600JD) was purchased from Canrd and the SWCNT (XFS30) was purchased from XFNANO. BPKC was prepared by ball milling the mixture of BP, Ketjenblack, and SWCNT with a mass ratio of 7/2.5/0.5 using the high energy ball milling machine for 10 h at 1000 rpm in a stainless steel jar purged with argon.^[1]^ The ZC was prepared by a solvothermal method based on previous literature.^[2,3]^ Specifically, imidazole (Im, Aladdin, 99.5%, 11.55 mmol, 785.4 mg) and benzimidazole (BIm, Aladdin, 98%, 1.66 mmol, 196 mg) were dissolved in N, N-dimethylformamide (DMF, 90 mL, Aladdin, 99.9%). Subsequently, Co(NO_3_)_2_·6H_2_O (4 mmol, Aladdin, 99.9%, 1164 mg) was dissolved in the above solution, and after stirring, the solution was heated at 130 °C for 3 days and the product was separated by centrifugation and dried under vacuum at 120 °C for 12 h. The ZG was synthesized by a melt-quenched method heating the finely ground ZC at 10 °C min^-1^ to 430 °C and calcining for 5 min under the atmosphere of argon, followed by cooling naturally to room temperature. The ZCP and ZGP were obtained by simply mixing the BP (15% of the total mass) with ZC and ZG respectively through ball milling at 500 rpm for 0.5 h. By mixing BPKC with ZC and subsequently applying the melt-quenched method with tube sealed properly, ZGPC15, ZGPC and ZGPC25 with BPKC content of 15%, 20% and 25% respectively can be obtained.

**Material Characterizations:** XRD measurement was implemented by using Bruker D8 Advance with Cu Kα radiation (λ=1.5406 Å) during the 2*θ* range of 10-80°. The DSC and TG measurements were performed at Ar atmosphere with a heating rate of 10 ºC min^-1^ from room temperature to 600 ºC using a simultaneous thermal analyzer (HITACHI STA200, Japan). The Raman analysis was conducted with a confocal Raman microscope (CRM) (Alpha300R, WITec GmbH, Germany) equipped with a TEM single-frequency laser (λ=532 nm). The FTIR spectra was obtained using Thermo Scientific Nicolet iS50 (USA) in the range of 400-4000 cm^-1^. The morphologies of the samples were observed through SEM (ZEISS GeminiSEM 360, Germany) and TEM (FEI Talos F200x, USA). XPS spectra were collected by using Thermo SCIENTIFIC Nexsa (USA) with Al Ka X-ray (1486.6 eV) at a pass energy of 50 eV. EPR test was carried out using Bruker EMXplus-6/1 (Germany), and the N2 adsorption-desorption isotherms were acquired with Micromeritics Tristar 3000 (USA). In situ TEM observation for the lithiation/delithiation of ZGPC was performed on a TEM (JEOL F200) operated at 200 kV equipped with a Nanofactory EP1000 TEM-STM holder. Practically, ZGPC was placed at one side of the holder, acting as working electrode. At the other side of the holder, a piece of Li metal was loaded on a Cu tip, serving as counter electrode. During sample transfer, a layer of Li_2_O was naturally formed on the surface of Li metal, acting as the solid electrolyte. The Li/Li_2_O and ZGPC were connected inside TEM facilitated by a piezoelectric manipulator. Then, a positive bias (+3 V) was applied on the Li/Li_2_O electrode to initiated the lithiation process of ZGPC. By reversing the applied voltage, the delithiation process of ZGPC can be observed.

**Cell Assembly and Electrochemical Characterizations:** The lithium storage performances were assessed with metallic lithium foil (diameter of 15.6 mm) as the counter electrode using coin-type cell CR2032. The working electrodes were fabricated by pasting well-mixed slurry consisting of active materials (80 wt%), conductive acetylene black (10 wt%) and polytetrafluoroethylene (PVDF, 10 wt%) on a copper foil substrate. Subsequently, the working electrodes were dried under vacuum at 100 ºC overnight and cut into circles with a diameter of 12 mm. The average mass loadings of the active materials on the working electrode were evaluated as around 1.3 mg cm^-2^. The cells were assembled under Argon in a glovebox with the electrolyte consists of 1 M LiPF_6_ in EC/DEC (1:1 vol%) and the Canrd 2500 membrane (diameter of 18.0 mm) as the separator. The GCD measurements were employed on the NEWARE battery test system (CT-4008Tn, China) at 25 ºC. CV curves were collected in the voltage range of 0.01-3.0 V at the scanning rate of 0.1 mV s^-1^, and EIS tests were carried out in the frequency range of 0.1 to 100 kHz on a CHI760E electrochemical workstation with an amplitude of 5 mV. The GITT was implemented in the potential range of 0.01-3.0 V at 0.1 A g^-1^ with 15-minute pulse current time and 120-minute rest time.

**Computational Methods:** Density Functional Theory (DFT) calculations were performed within the Generalized Gradient Approximation (GGA) framework, employing the Perdew-Burke-Ernzerhof (PBE) exchange-correlation functional,^[4–7]^ as implemented in the Vienna Ab initio Simulation Package (VASP). A plane-wave basis set with an energy cutoff of 350 eV was used. The Brillouin zone was sampled using a 1 × 1 × 1 Gamma k-point grid. Geometry optimizations were conducted until the atomic forces converged to less than 0.05 eV/Å. To simulate the migration pathways and energy barriers of lithium ions (Li⁺) in ZC and BP/CoP@ZIF heterojunctions, the climbing-image nudged elastic band (CI-NEB) method was employed.^[8,9]^ The force convergence criterion was set to 0.05 eV/Å, with a climbing image algorithm (LCLIMB = .TRUE.) and a spring constant of -5 eV/Å².

**Kinetic Analysis through the CV results:**

The capacitive and diffusion contribution at various scan rates can be quantified according to the equation:

$i\left( v \right)=k_{1}v+k_{2}v^{1/2}$ (1)

Where, $i$ is the peak current, $v$ is the scan rate, $k_{1}v$ represents capacitive contribution and $k_{2}v^{1/2}$ corresponds to diffusion-controlled contribution.

**Fitting of the Electrochemical Impedance Spectroscopy (EIS):**

The Nyquist plots of the anodes before cycling are composed of one depressed semicircle during the high frequency region and an inclined line in the low frequency range, referring to the charge transfer resistance (R_ct_) and Warburg resistance, respectively. After cycling, the depressed semicircles in the curves split into two, while the one in the higher frequency region appears to be the impedance of solid electrolyte interface layer (R_s_). The EIS fitting was based on corresponding equivalent circuit models (Figure S18, Supporting Information), and Table S2, Supporting Information, displays the calculated values.

**Calculation of the Activation Energies (E_a_)**:

EIS measurements at different temperatures were conducted, and E_a_ were calculated based on the following Arrhenius equation:

$\ln\left( \frac{1}{R_{ct}} \right)=-\frac{1}{T}\cdot\frac{E_{a}}{R}+\ln A_{0}$ (2)

Where, R_ct_ is the charge transfer impedance, T is temperature in Kelvin, R is the gas constant, and A is Arrhenius factor, which indicates the rate of collision and the fraction of collisions with the proper orientation for the reaction to occur.

**Calculation of Li^+^ Diffusion Coefficient through GTTT Results:**

GITT were implemented and the diffusion coefficients for lithium ions (D_Li_^+^) are calculated to examine the reaction dynamics of the electrodes according to the following equation:

$D^{\mathrm{GITT}}=\frac{4}{\pi\tau}\left( \frac{mV_{M}}{MS} \right)^{2}\left( \frac{\Delta E_{s}}{\Delta E_{\tau}} \right)^{2}$ (3)

where m, V_M_ and M represent the mass, molar volume and the molar mass of the active materials respectively. S refers to the electrode area while τ denotes the duration of the pulse. ∆E_s_ represents voltage drop caused by the current pulse during the equilibrium state, and ∆E_τ_ represents the voltage change caused by the constant current charge/discharge processes.


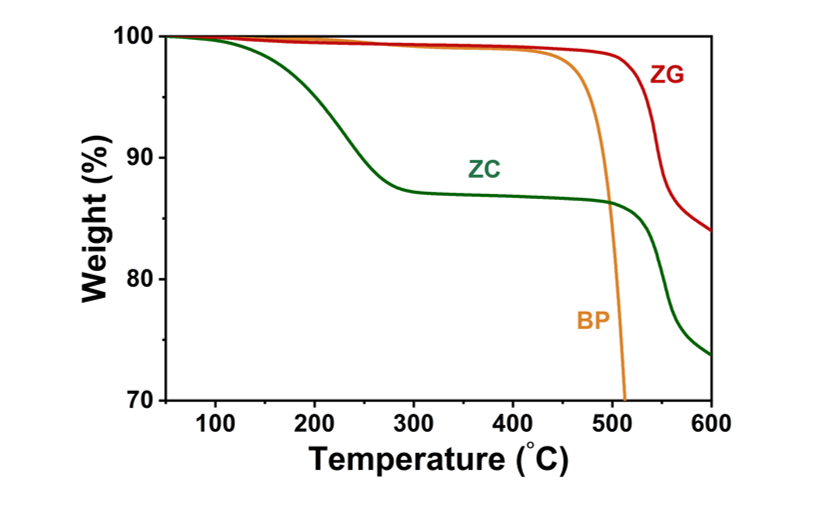


1. TG analysis of ZC, ZG and BP with a heating rate of 10 ℃ min^-1^ at Ar atmosphere.


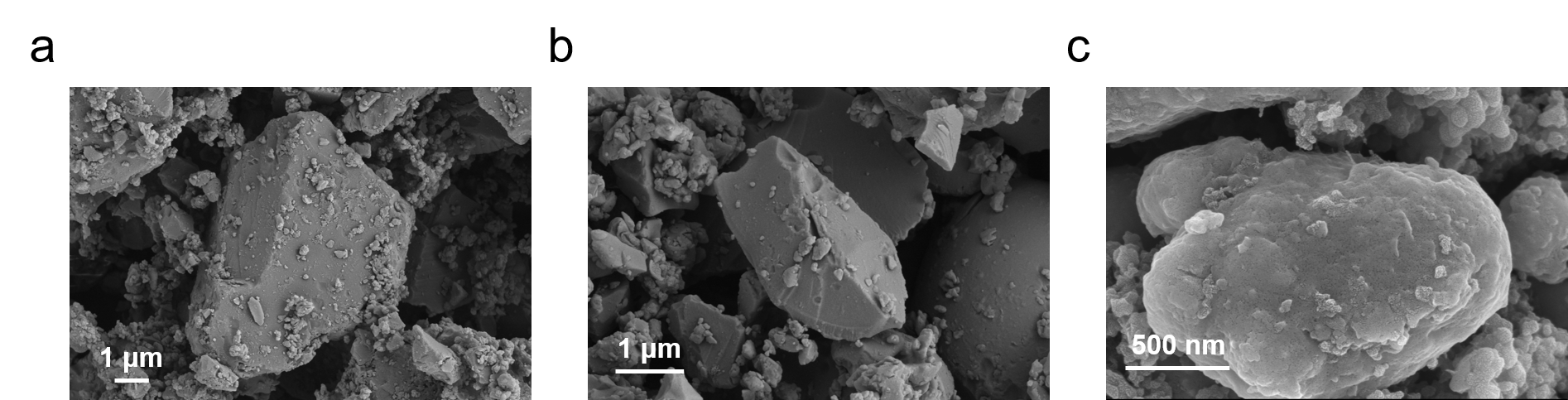


1. Typical SEM images of ZC (a), ZG (b) and ZGPC (c).


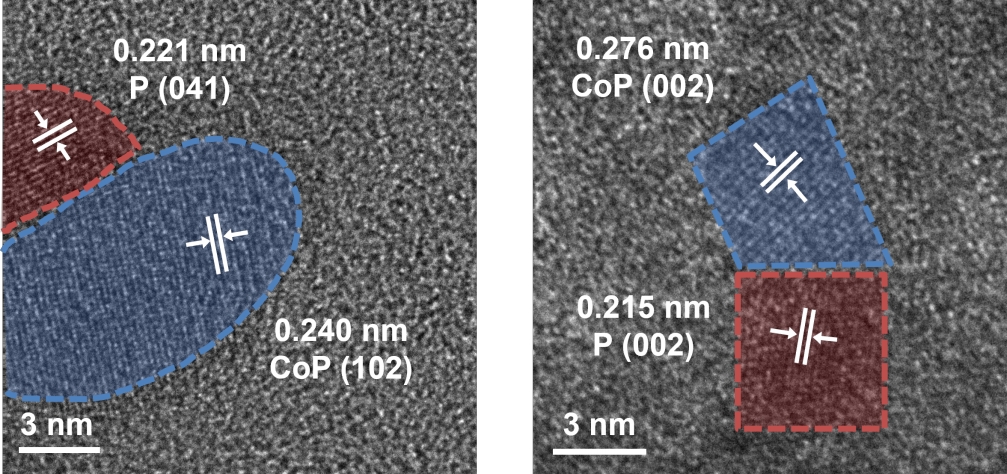


1. HRTEM images for ZGPC.


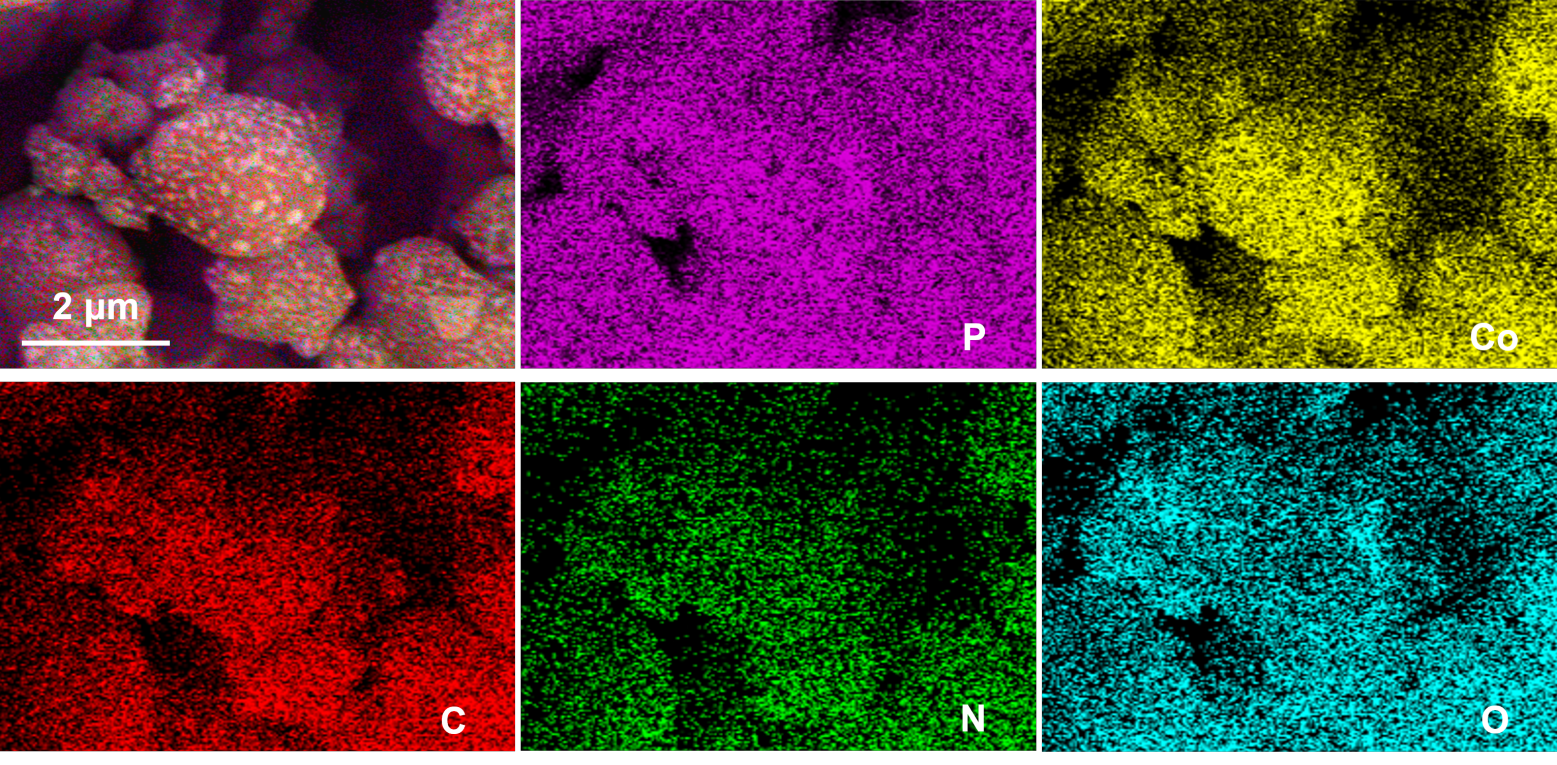


1. EDS elemental mappings of P, Co, C, N and O for ZGPC.


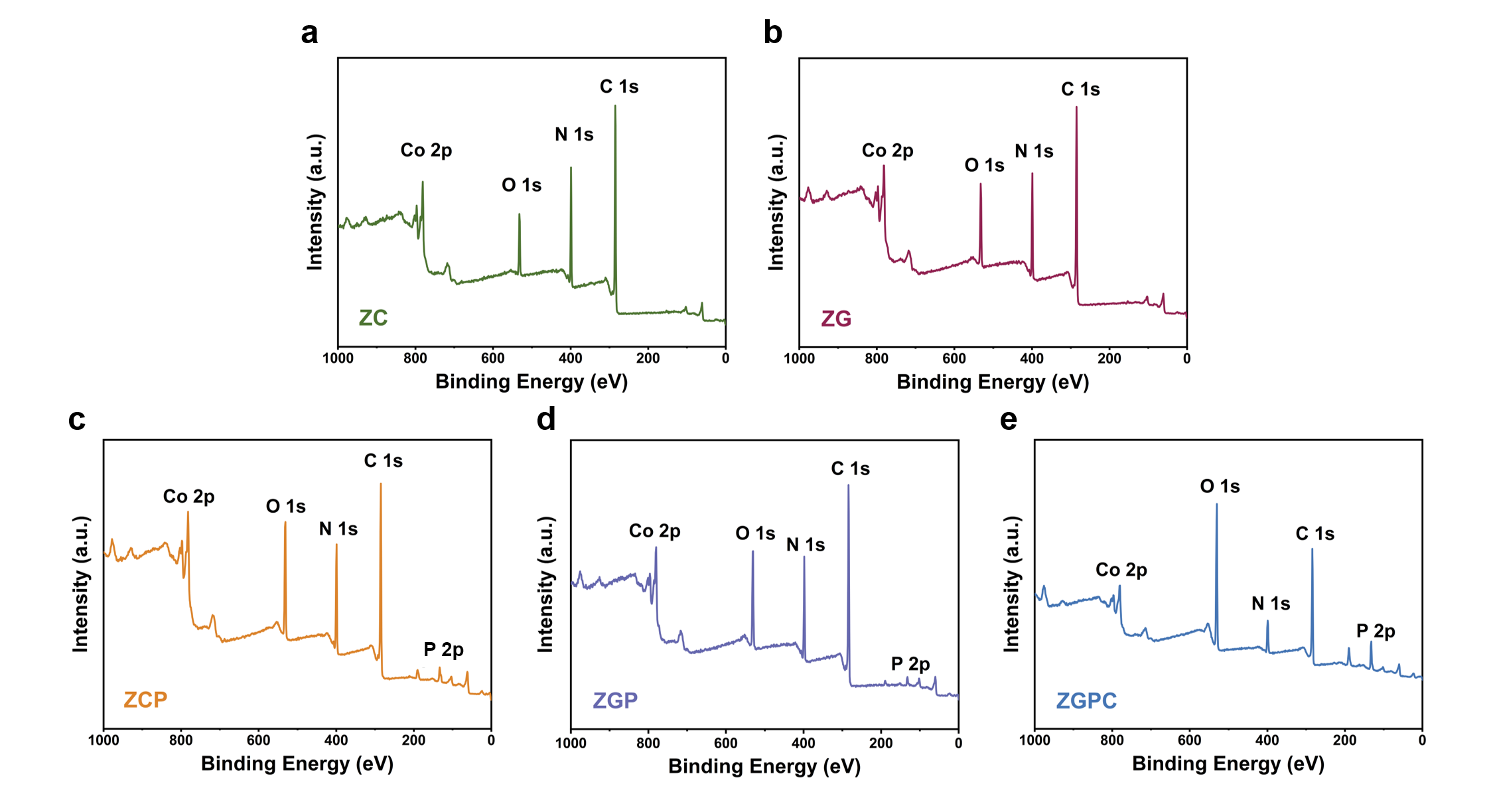


1. Full XPS survey spectra for ZC(a), ZG(b), ZCP(c), ZGP(d) and ZGPC(e).


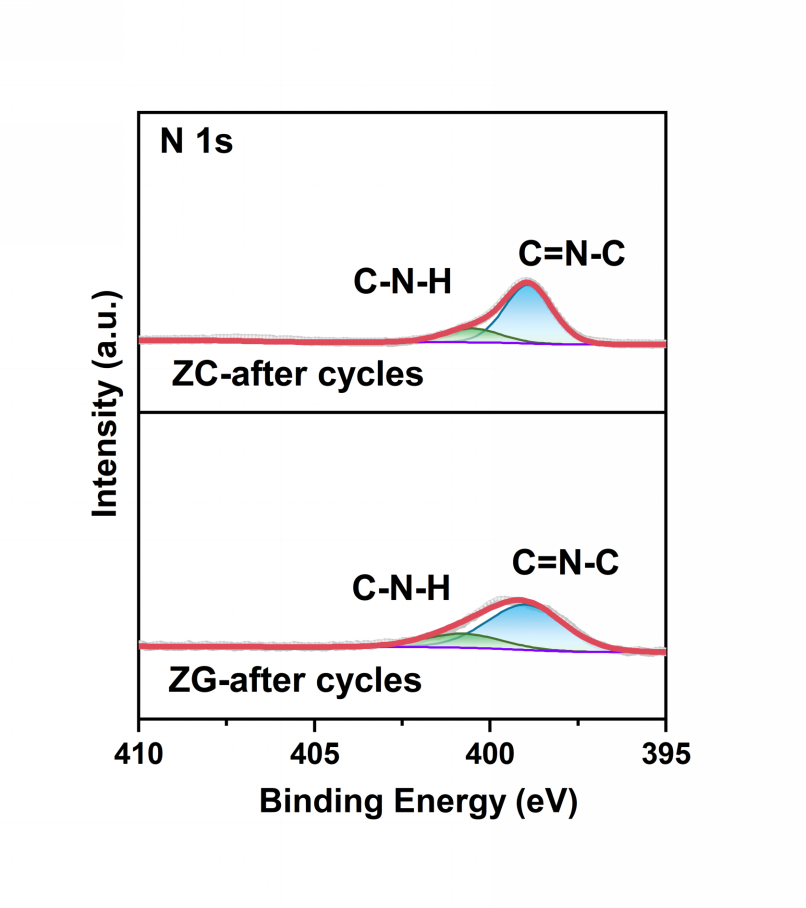


1. High resolution spectra of N 1s for ZC and ZG after 50 cycles.


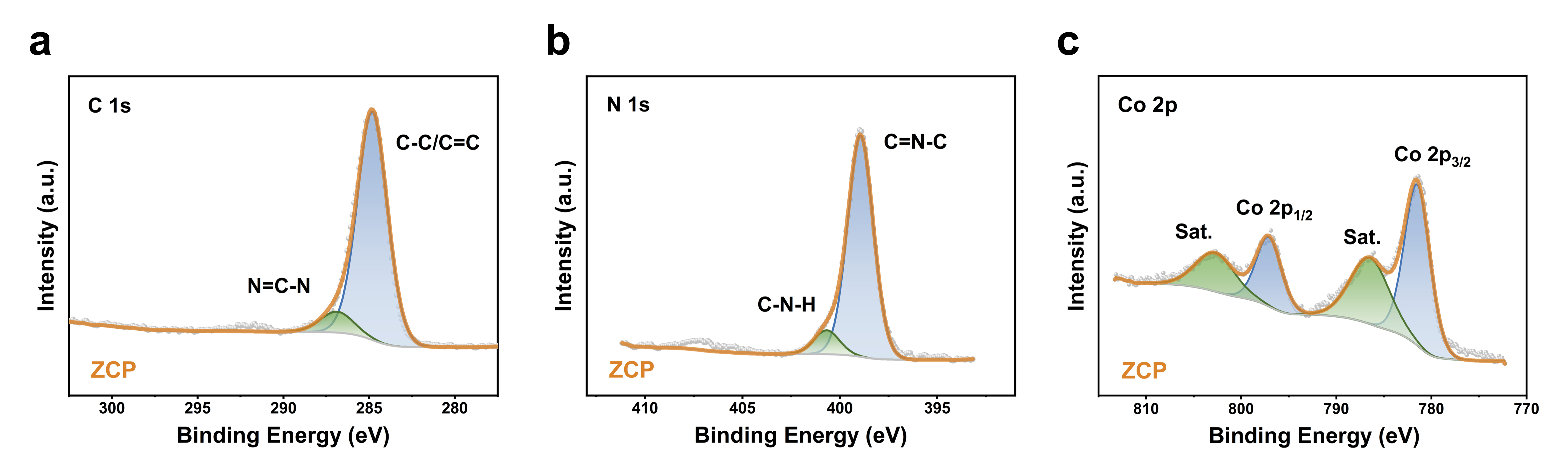


1. High resolution spectra of C 1s (a), N 1s (b) and Co 2p (c) for ZCP.


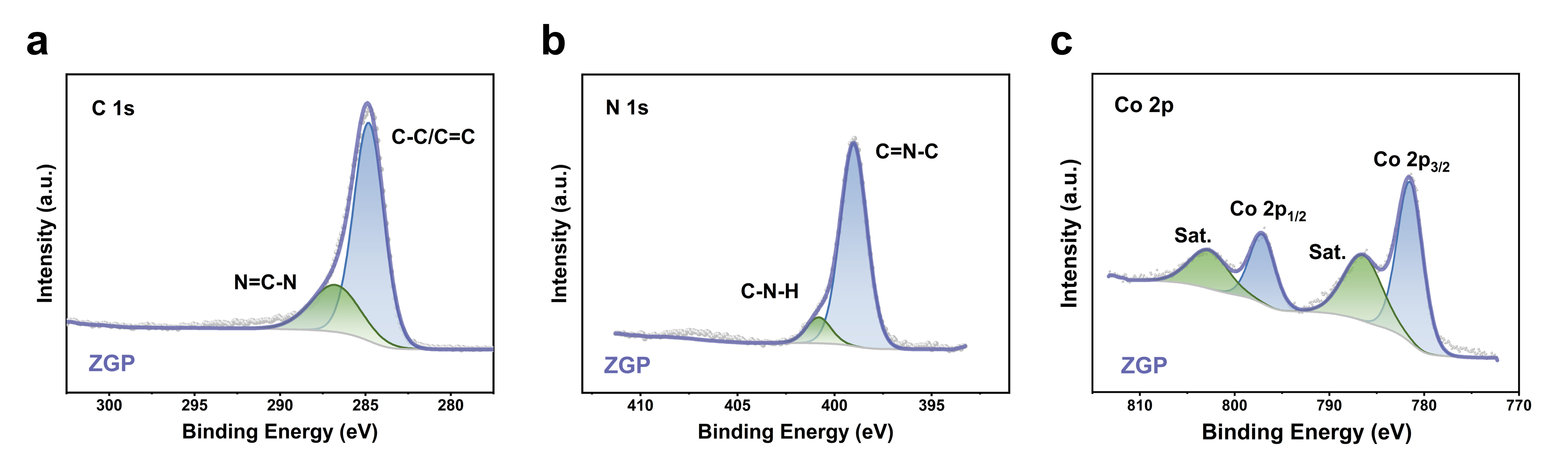


1. High resolution spectra of C 1s (a), N 1s (b) and Co 2p (c) for ZGP.


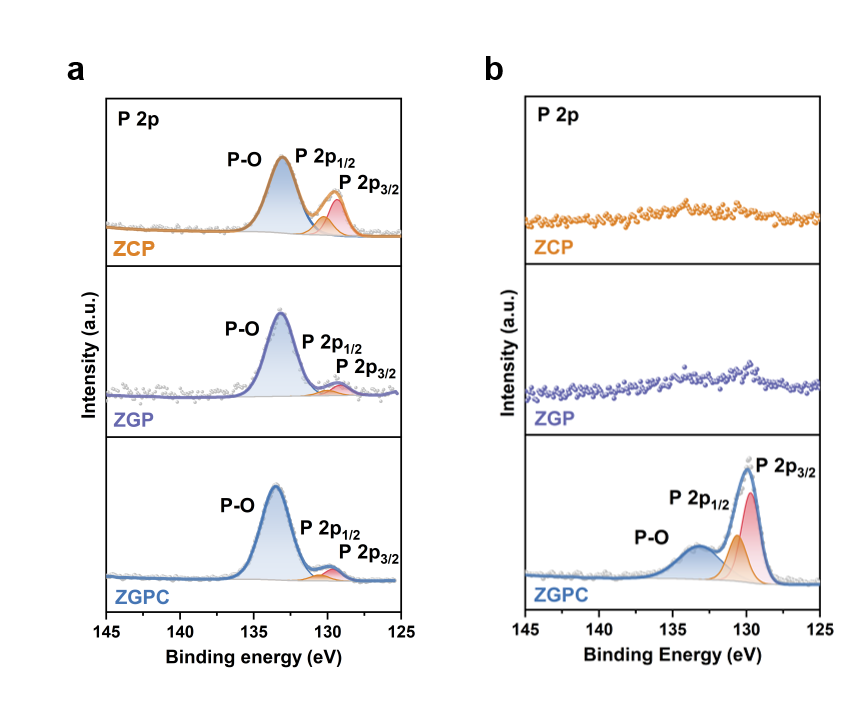


1. High resolution spectra of P 2p (a) and in depth XPS in P 2p core levels with 60 s duration of etching (b) for ZCP, ZGP and ZGPC.


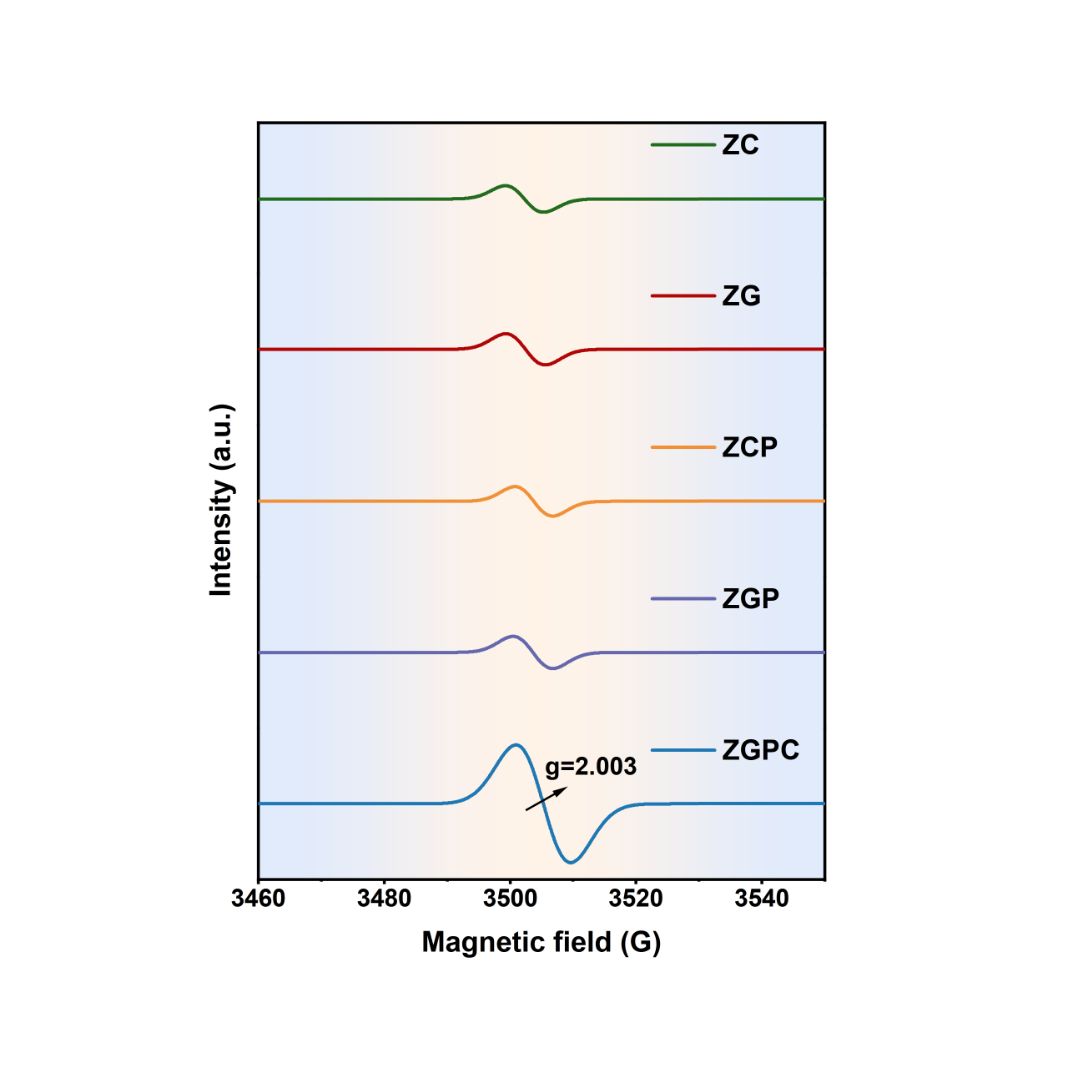


1. EPR spectra of ZC, ZG, ZCP, ZGP and ZGPC.


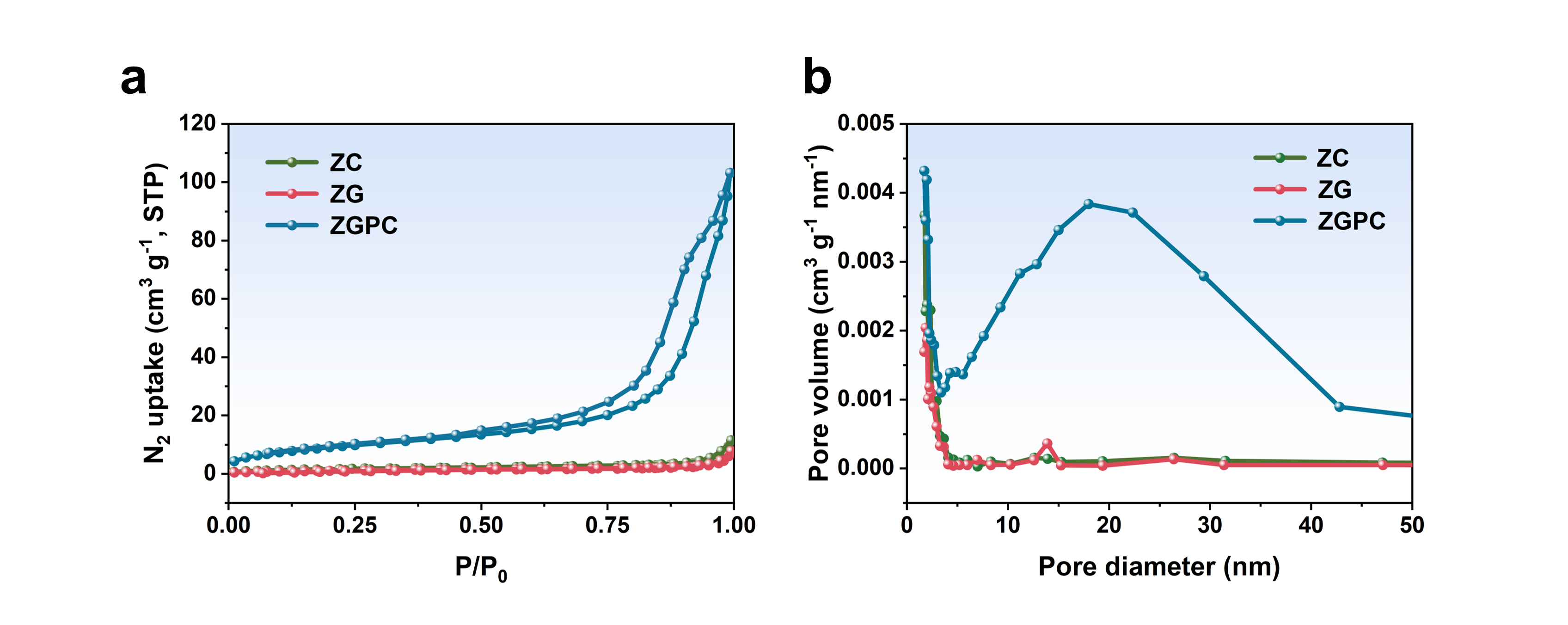


1. N_2_ adsorption/desorption isothermal curves (a) and pore size distribution (b) of ZC, ZG and ZGPC.


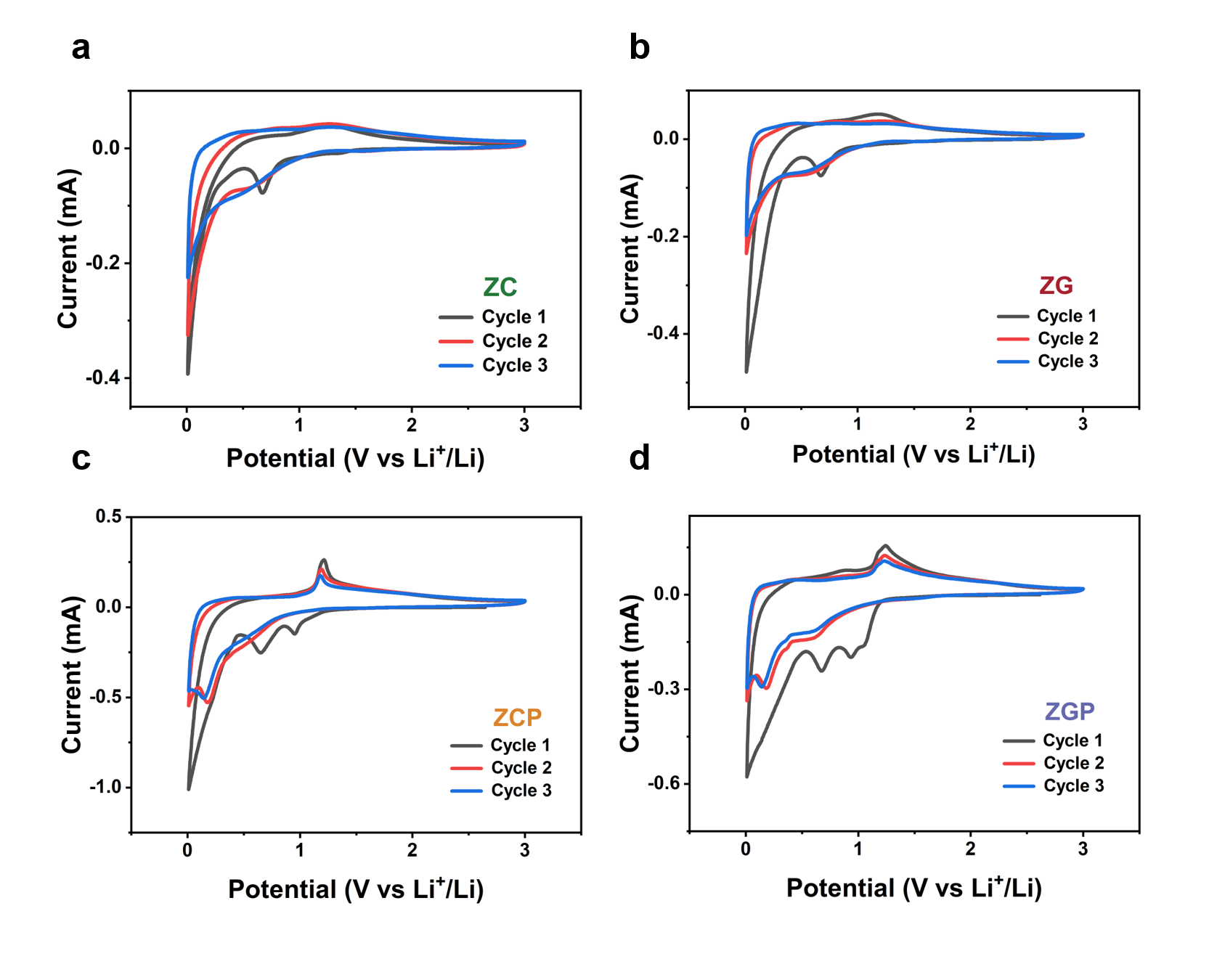


1. CV curves of ZC (a), ZG (b), ZCP(c) and ZGP (d) within the range of 0.01-3.0 V at a scan rate of 0.1 mV s^-1^.


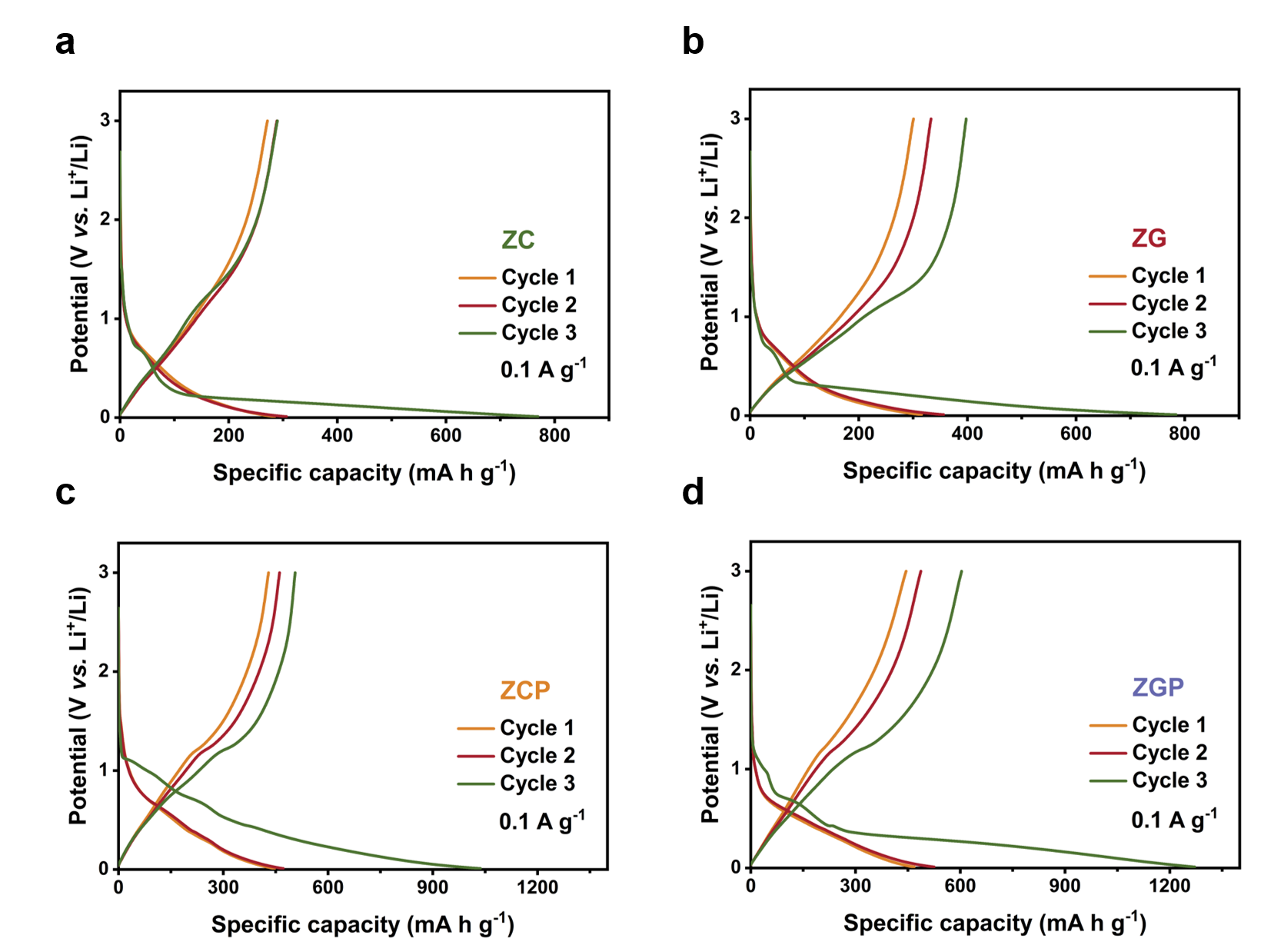


1. The GCD profiles of ZC (a), ZG (b), ZCP(c) and ZGP (d) at 0.1 A g^−1^ for the first 3 cycles.


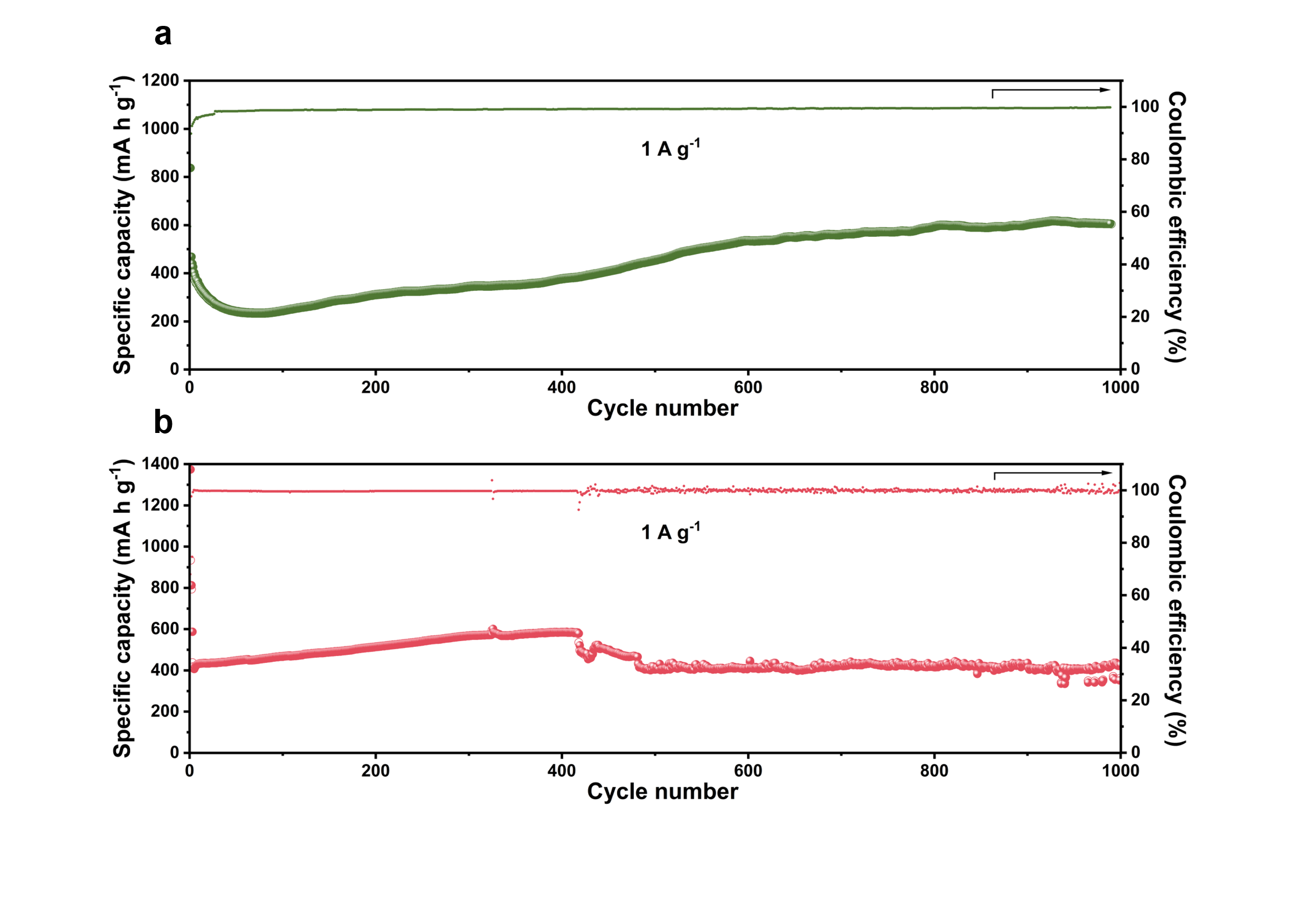


1. To investigate the performance under different concentrations of BPKC, we also tested the ZGPC15 and ZGPC25 samples where the BPKC contents are 15% and 25%, respectively. Cycling performance of ZGBP15 (a) and ZGPC25 (b) at 1 A g^−1^ using electrolyte consisting of 1 M LiPF_6_ in EC/DEC are plotted. ZGPC15 with less BP content shows lower capacity at the early stage, while ZGPC25 delivers the highest initial reversible capacity of 934.52 mAh g^−1^. However, as more BP introduced into the composite, the ZIF glass structure of ZGPC25 is unable to accommodate all BP and the cycling thereby becomes unstable after about 400 cycles, implying a higher degree of uncontrollable volume change of BP without proper confinement of ZG.


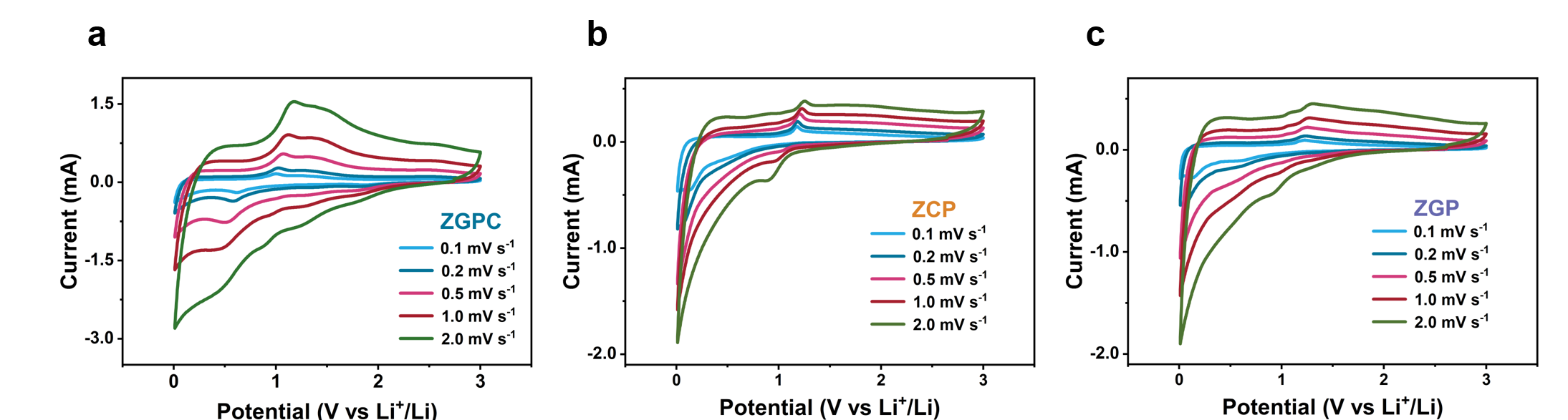


1. CV curves of ZGPC (a), ZCP (b) and ZGP (c) within the range of 0.01-3.0 V at different scan rates.


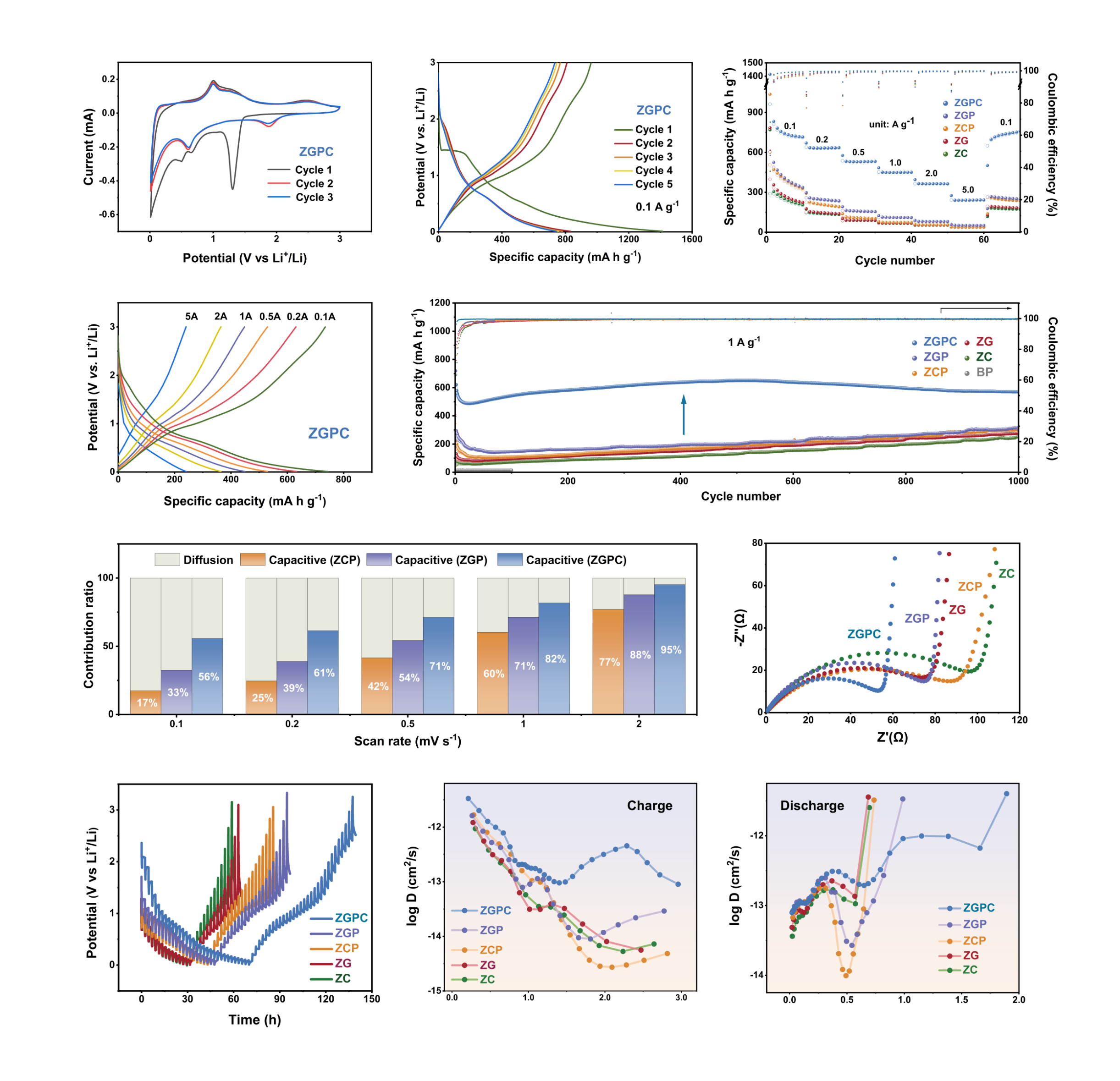


1. Contribution ratio of diffusion-controlled and capacitive-controlled calculated from CV curves under different scan rates of ZCP, ZGP and ZGPC.


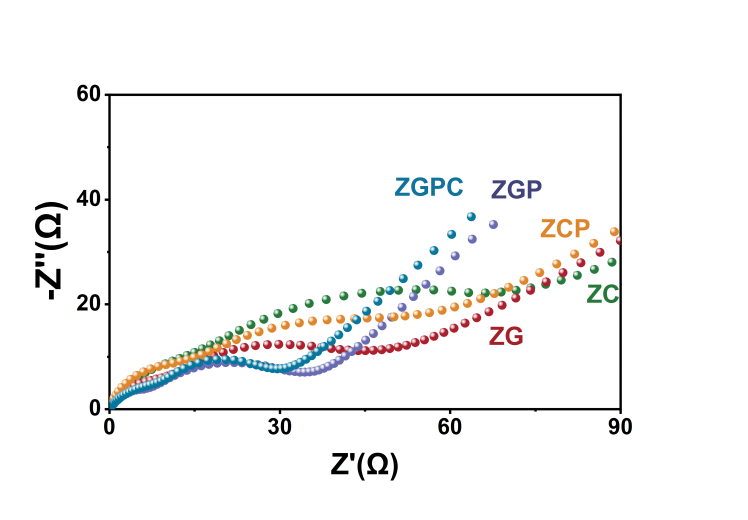


1. Nyquist plots obtained from electrochemical impedance spectra within the frequency range from 0.01 to 100 kHz with an amplitude of 10 mV after 500 cycles.


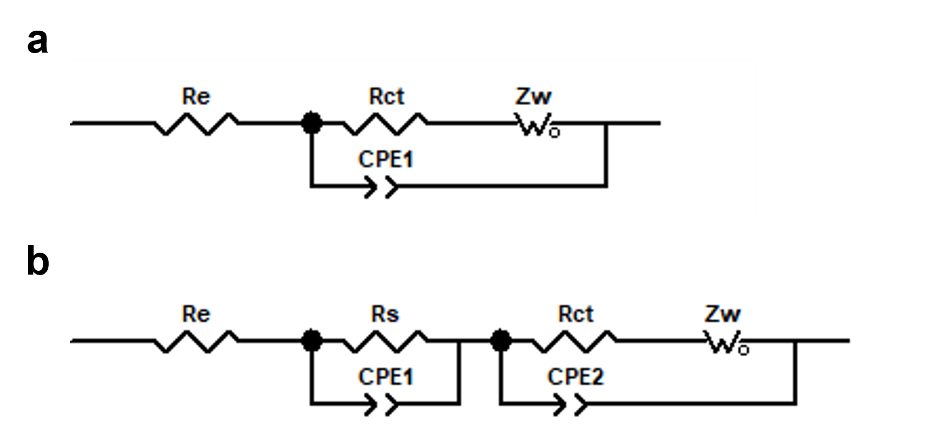


1. Equivalent circuit model for fitting Nyquist plots before (a) and after (b) cycling.


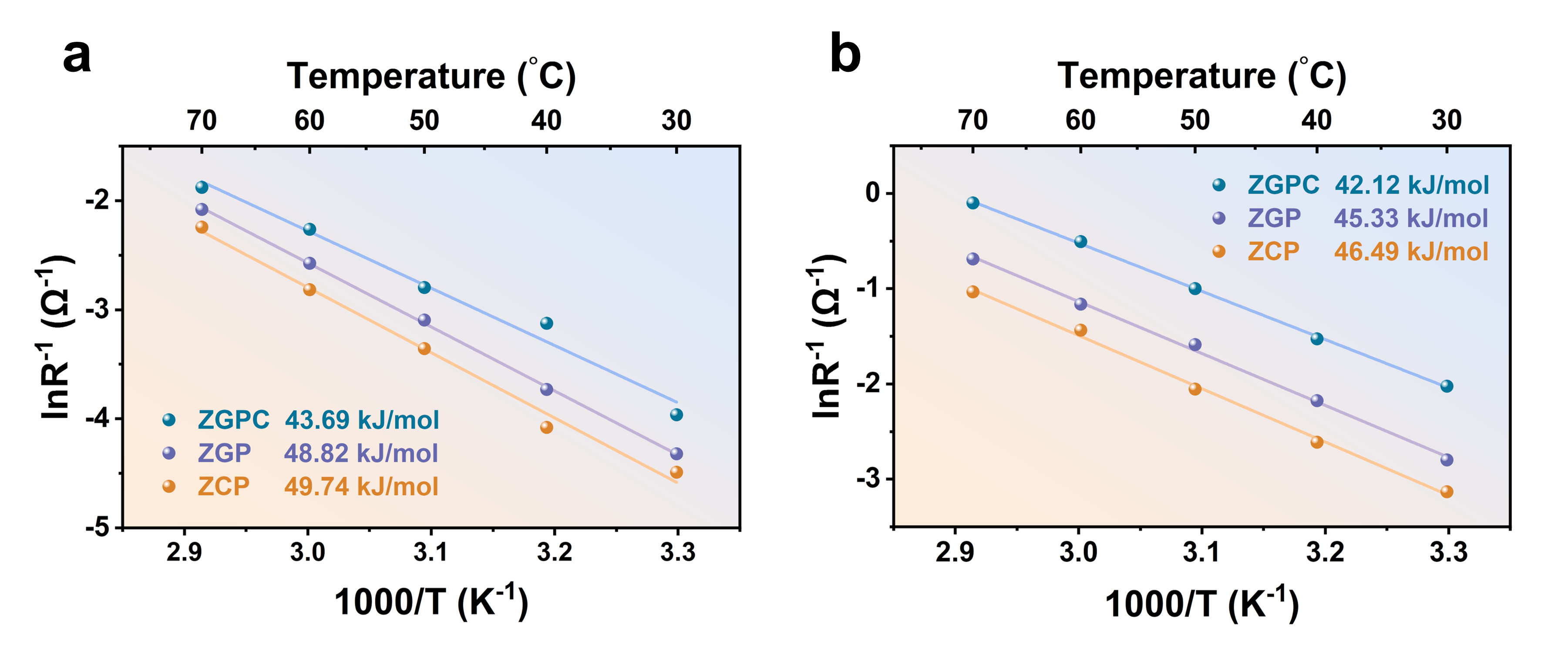


1. Arrhenius plots of impedance versus temperature for ZCP, ZGP and ZGPC before (a) and after 10 cycles (b) with the calculated values of Ea. Based on the Arrhenius formula, the activation energies of the cells with ZCP, ZGP and ZGPC anodes are calculated as 43.69, 48.82 and 49.74 KJ mol^−1^ before cycling and 42.12, 45.33 and 46.49 KJ mol^−1^ after cycling respectively.


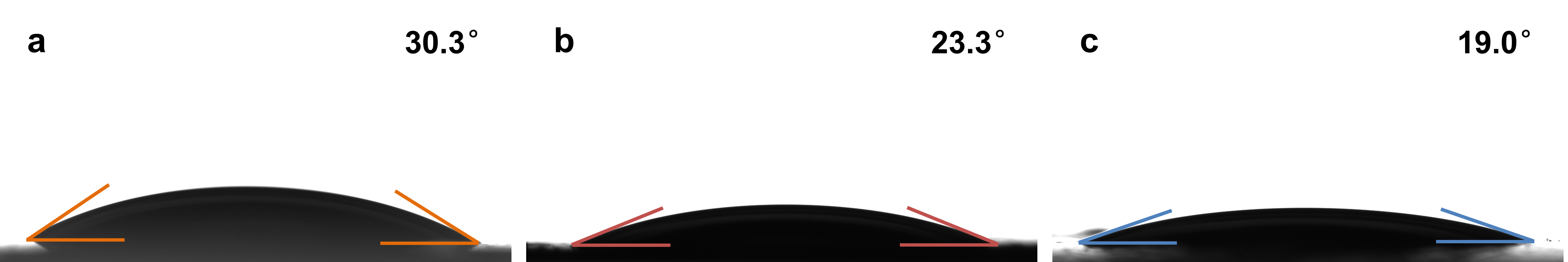


1. The contact angles of BP, BPKC and ZGPC with the electrolyte of 1 M LiPF_6_ in EC/DEC (1:1 vol%).


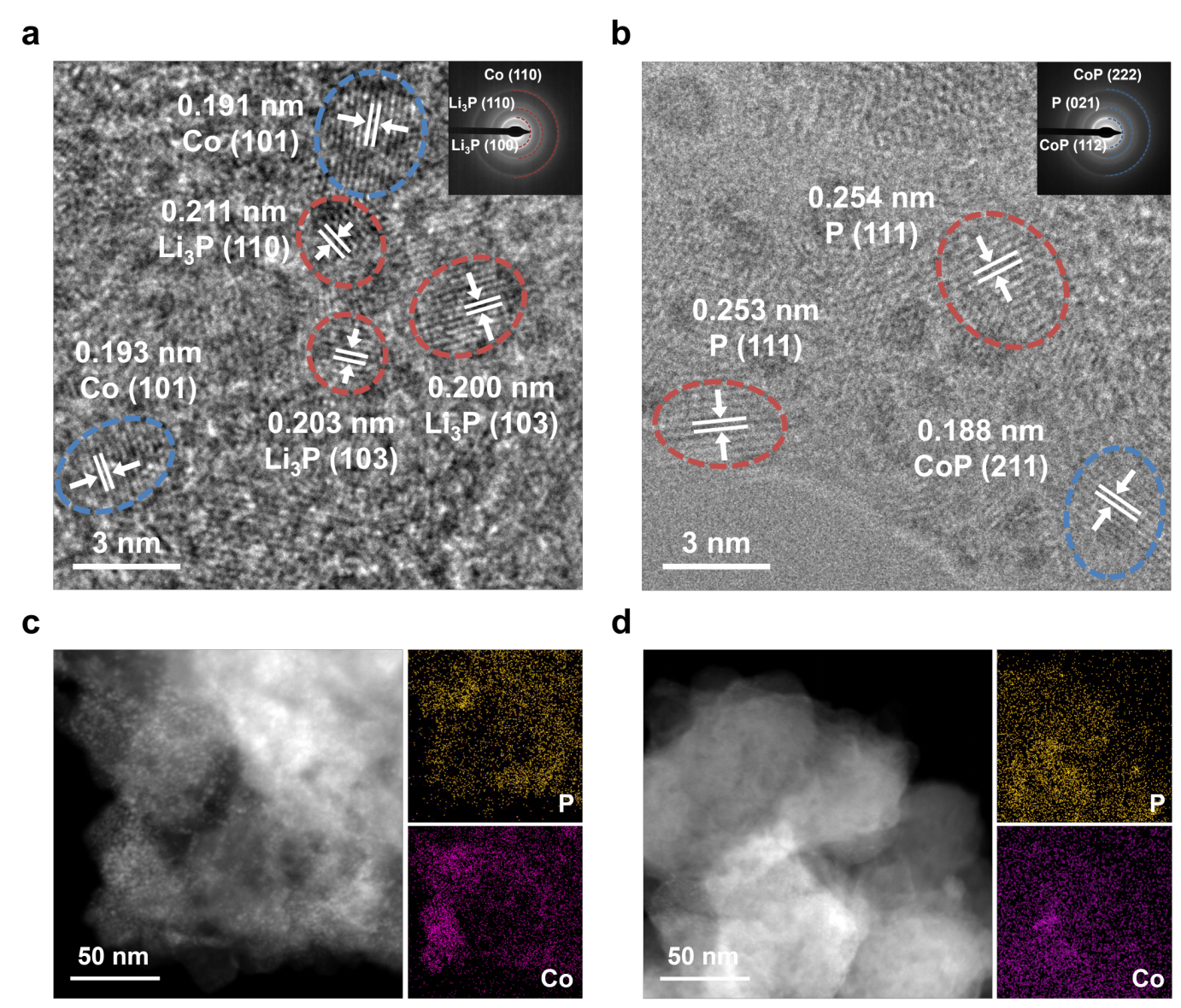


1. HRTEM image and the selected area electron diffraction (SAED) patterns for ZGPC at 0.2 V (a) and at 2.8 V (b). From the HRTEM images, some crystalline structures exhibit inside the disordered structure of ZIF glass at both the discharged and charged state, verifying the encapsulation of products in ZIF glass. At 0.2 V, we could index the spacing lattice of 0.200 and 0.203 nm to the (103) plane of Li_3_P phase, 0.211 nm to the (110) plane of Li_3_P phase, 0.191 and 0.193 nm to the (101) plane of Co phase, while in the SAED pattern, the typical diffraction ring of (110) and (100) planes of Li_3_P and the (110) plane of Co are observed. At 2.8 V, the spacing lattice of 0.253 and 0.254 nm can be ascribed to (111) plane of phosphorus with the spacing lattice of 0.188 nm assigned to (211) plane of CoP, which confirms the reversible formation of BP and CoP. The typical diffraction ring of (222) and (112) planes of CoP and the (021) plane of phosphorus can be detected in the SAED pattern.


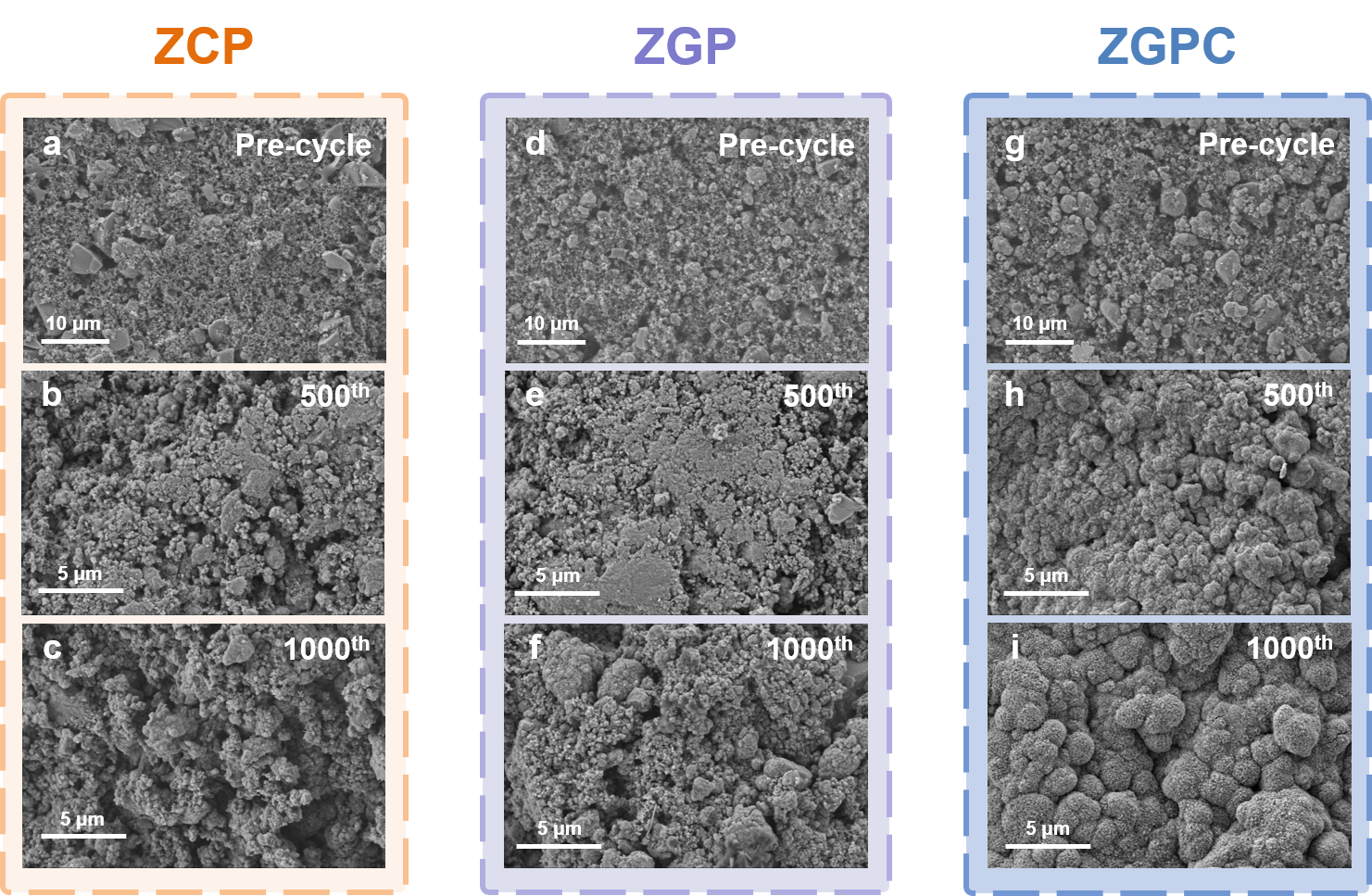


1. The SEM images of ZCP (a-c), ZGP (d-f) and ZGPC (g-i) anodes before cycling, after 500 cycles and after 1000 cycles at a current density of 1 A g^-1^.


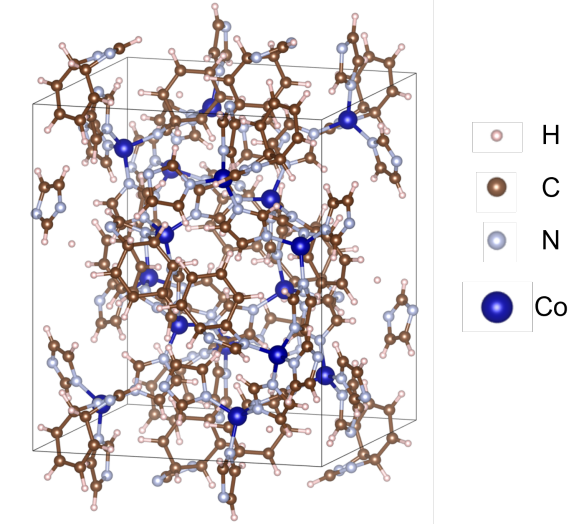


1. The simulated configuration of ZC for DFT calculations.


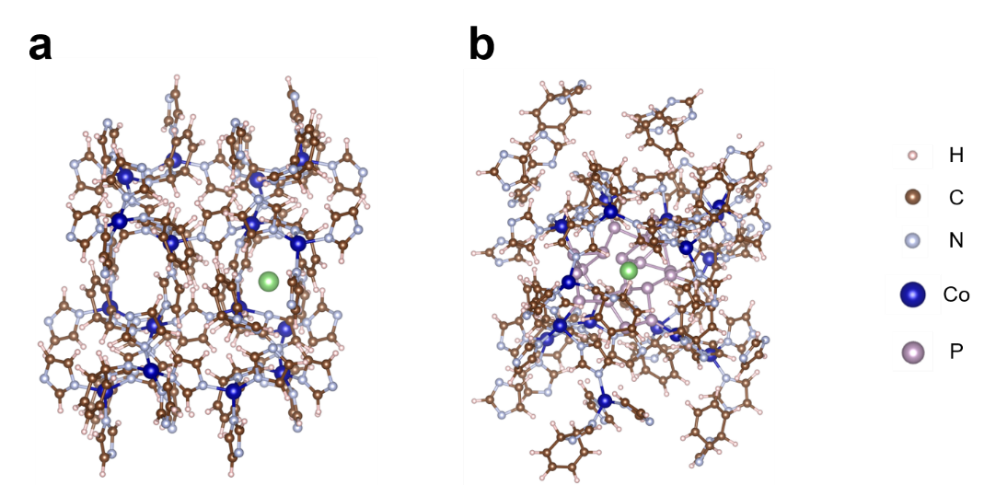


1. Binding sites for lithium storage in ZC (a) and BP/CoP@ZC (b).


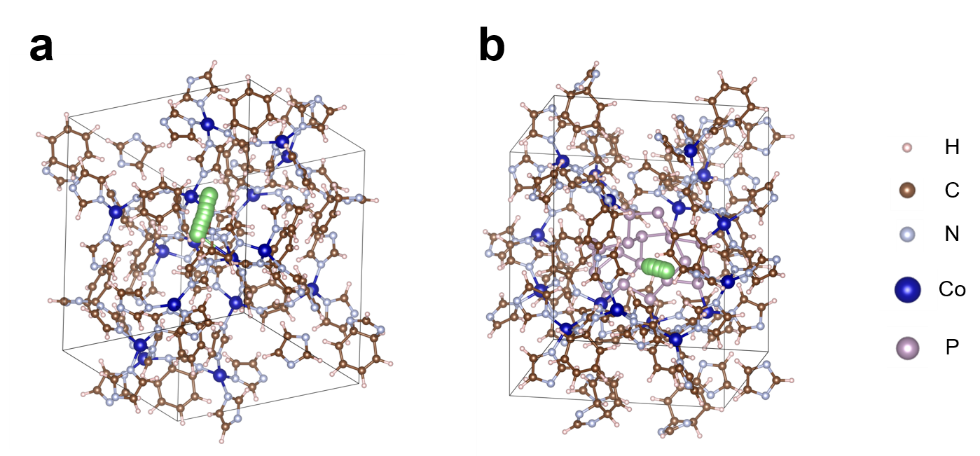


1. Migration path for lithium diffusion in ZC (a) and BP/CoP@ZC (b).

Table S1. The fractions of C=N-C, C-N-H of ZC and ZG before and after cycling, which were obtained from the high resolution spectra of N 1s presented in Figure 2 and Figure S8.

|  | **C=N-C (%)** | **C-N-H (%)** | **Ratio**  **(C=N-C/C-N-H)** |
| --- | --- | --- | --- |
| **ZC** | 89.3 | 10.7 | 8.35 |
| **ZG** | 88.5 | 11.5 | 7.70 |
| **ZC-after cycles** | 78.1 | 21.9 | 3.57 |
| **ZG-after cycles** | 75.8 | 24.2 | 3.13 |

Table S2. Comparing of key performance metrics of the ZGPC with other MOF glass-based anode materials for LIBs.

|  | **Rate**  **(A g^-1^)** | **Rate Capacity (mAh g^-1^)** | **ICE (%)** | **Cycle number** | **Capacity variation (%)** | **R_ct_ (Ω)** |
| --- | --- | --- | --- | --- | --- | --- |
| **This work (ZGPC)** | 5 | 245 | 68 | 1000 | 30 | 60 |
| **ZIF crystal (Ref. 31)** | 1 | ∼15 | ∼45 | 1000 | 94 | 258 |
| **ZIF glass (Ref. 31)** | 1 | ∼35 | ∼30 | 1000 | Over 200 | 218 |
| **SiZC (Ref. 32)** | 5 | ∼10 | ∼24 | 500 | Over 200 | 290 |
| **SiZGC (Ref. 32)** | 5 | ∼10 | ∼62 | 500 | Over 200 | 405 |

Table S3. Cycling performance comparison of ZGPC with recently reported phosphorus-based anode materials for LIBs.

|  | **Current Density** | **Cycle number** | **Capacity retention (%)** |
| --- | --- | --- | --- |
| **Ref. 52** | 1 A g^-1^ | 1000 | 50.7 |
| **Ref. 53** | 2 A g^-1^ | 800 | 61.9 |
| **Ref. 54** | 0.78 A g^-1^ | 300 | 65.7 |
| **Ref. 55** | 1.281 A g^-1^ | 300 | 61.0 |
| **Ref. 56** | 1 A g^-1^ | 545 | 87.0 |
| **Ref. 57** | 1.3 A g^-1^ | 500 | 87.0 |
| **Ref. 58** | 1 mA cm^-2^ | 140 | 71.4 |
| **Ref. 59** | 1.3 A g^-1^ | 650 | 81.9 |
| **Ref. 60** | 0.4 A g^-1^ | 1000 | 86.0 |
| **Ref. 61** | 1 A g^-1^ | 600 | 74.0 |
| **Ref. 62** | 1 A g^-1^ | 100 | 85.3 |
| **This work (ZGPC)** | 1 A g^-1^ | 1000 | 98.6 |

Table S4. Calculated impedance values from EIS equivalent circuit model.

|  | **R_e_ (Ω)** | **R_s_ (Ω)** | **R_ct_ (Ω)** |
| --- | --- | --- | --- |
| **ZC** | 4.25 | / | 112.80 |
| **ZG** | 4.64 | / | 86.81 |
| **ZCP** | 2.41 | / | 94.33 |
| **ZGP** | 5.53 | / | 83.70 |
| **ZGPC** | 5.37 | / | 60.78 |
| **ZC-after cycles** | 6.17 | 11.71 | 43.11 |
| **ZG-after cycles** | 6.66 | 9.59 | 29.60 |
| **ZCP-after cycles** | 6.08 | 12.64 | 35.27 |
| **ZGP-after cycles** | 6.12 | 5.32 | 26.41 |
| **ZGPC-after cycles** | 8.75 | 12.09 | 15.62 |

**Reference**

[1] G.-L. Xu, Z. Chen, G.-M. Zhong, Y. Liu, Y. Yang, T. Ma, Y. Ren, X. Zuo, X.-H. Wu, X. Zhang, K. Amine, *Nano Lett.* **2016**, *16*, 3955.

[2] L. Frentzel-Beyme, M. Kloß, R. Pallach, S. Salamon, H. Moldenhauer, J. Landers, H. Wende, J. Debus, S. Henke, *J. Mater. Chem. A* **2019**, *7*, 985.

[3] C. Gao, Z. Jiang, S. Qi, P. Wang, L. R. Jensen, M. Johansen, C. K. Christensen, Y. Zhang, D. B. Ravnsbæk, Y. Yue, *Adv. Mater.* **2022**, *34*, 2110048.

[4] G. Kresse, J. Furthmüller, *Comput. Mater. Sci* **1996**, *6*, 15.

[5] P. Hohenberg, W. J. P. R. Kohn, *Phys. Rev* **1964**, *136*, B864.

[6] W. Kohn, L. J. Sham, *Phys. Rev.* **1965**, *140*, A1133.

[7] A. I. Liechtenstein, V. I. Anisimov, J. Zaanen, *Phys. Rev. B* **1995**, *52*, R5467.

[8] G. Henkelman, B. P. Uberuaga, H. Jónsson, *J. Chem. Phys.* **2000**, *113*, 9901.

[9] D. Sheppard, R. Terrell, G. Henkelman, *J. Chem. Phys.* **2008**, *128*, 134106.
